# A quality indicator set for rehabilitation services for people with rheumatic and musculoskeletal diseases demonstrates adequate responsiveness in a pre–post evaluation

**DOI:** 10.1186/s12913-021-06164-2

**Published:** 2021-02-20

**Authors:** Anne-Lene Sand-Svartrud, Gunnhild Berdal, Maryam Azimi, Ingvild Bø, Turid Nygaard Dager, Siv Grødal Eppeland, Guro Ohldieck Fredheim, Anne Sirnes Hagland, Åse Klokkeide, Anita Dyb Linge, Kjetil Tennebø, Helene Lindtvedt Valaas, Ann Margret Aasvold, Hanne Dagfinrud, Ingvild Kjeken

**Affiliations:** 1 National Advisory Unit on Rehabilitation in Rheumatology, Diakonhjemmet Hospital, PO Box 23, Vinderen, N-0319 Oslo, Norway; 2 Diakonhjemmet Hospital, PO Box 23, Vinderen, N-0319 Oslo, Norway; 3 Hospital for Rheumatic Diseases Lillehammer, Margrethe Grundtvigs veg 6, N-2609 Lillehammer, Norway; 4 Sørlandet Hospital Arendal, PO Box 416, Lundsiden, N-4604 Kristiansand, Norway; 5 Vikersund Rehabilitation Center, Haaviks vei 25, N-3370 Vikersund, Norway; 6 Hospital for Rheumatic Diseases Haugesund, PO Box 2175, N-5504 Haugesund, Norway; 7 Rehabilitering Vest Rehabilitation Center, PO Box 2175, N-5504 Haugesund, Norway; 8 Muritunet Rehabilitation Center, Grandedata 58, N-6210 Valldal, Norway; 9 Valnesfjord Health Sports Center, Østerkløftveien 249, N-8215 Valnesfjord, Norway; 10 Meråker Rehabilitation Center, Østigardsveien 24, N-7530 Meråker, Norway

**Keywords:** Rehabilitation, Musculoskeletal disease, Quality indicator, Health care, Responsiveness

## Abstract

**Background:**

Quality of care is gaining increasing attention in research, clinical practice, and health care planning. Methods for quality assessment and monitoring, such as quality indicators (QIs), are needed to ensure health services in line with norms and recommendations. The aim of this study was to assess the responsiveness of a newly developed QI set for rehabiliation for people with rheumatic and musculoskeletal diseases (RMDs).

**Methods:**

We used two yes/no questionnaires to measure quality from both the provider and patient perspectives, scored in a range of 0–100% (best score, 100%). We collected QI data from a multicenter stepped-wedge cluster-randomized controlled trial (the BRIDGE trial) that compared traditional rehabilitation with a new BRIDGE program designed to improve quality and continuity in rehabilitation. Assessment of the responsiveness was performed as a pre–post evaluation: Providers at rehabilitation centers in Norway completed the center-reported QIs (*n* = 19 structure indicators) before (T1) and 6–8 weeks after (T2) adding the BRIDGE intervention. The patient-reported QIs comprised 14 process and outcomes indicators, measuring quality in health services from the patient perspective. Pre-intervention patient-reported data were collected from patients participating in the traditional program (T1), and post-intervention data were collected from patients participating in the BRIDGE program (T2). The patient groups were comparable. We used a construct approach, with a priori hypotheses regarding the expected direction and magnitude of PR changes between T1 and T2. For acceptable responsivess, at least 75% of the hypotheses needed to be confirmed.

**Results:**

All eight participating centers and 82% of the patients (293/357) completed the QI questionnaires. Responsiveness was acceptable, with 44 of 53 hypotheses (83%) confirmed for single indicators and 3 of 4 hypotheses (75%) confirmed for the sum scores.

**Conclusion:**

We found this QI set for rehabilitation to be responsive when applied in rehabilitation services for adults with various RMD conditions. We recommend this QI set as a timely method for establishing quality-of-rehabilitation benchmarks, promoting important progress toward high-quality rehabilitation, and tracking trends over time.

**Trial registration:**

The study is part of the larger BRIDGE trial, registered at ClinicalTrials.gov (Identifier: NCT03102814).

**Supplementary Information:**

The online version contains supplementary material available at 10.1186/s12913-021-06164-2.

## Background

In recent decades, new knowledge has led to earlier diagnosis and more effective pharmacological and surgical treatment for people with rheumatic and musculoskeletal diseases (RMDs) [1]. Nevertheless, many in this population experience a suboptimal effect of such treatments and need rehabilitation services in primary and secondary health care [2, 3]. Unmet needs are often related to persistent or fluctuating symptoms such as pain, fatigue, stiffness, and joint swelling [4] and can be reflected in individual rehabilitation goals. These goals may span several areas, including physical or mental functioning, personal activities of daily living, social participation, education, and work productivity [5–7].

The wide range of rehabilitation needs calls for individualized interventions, a multidisciplinary approach, and coordination across levels of care to ensure continuity in rehabilitation pathways. Furthermore, sufficient time is needed for individuals to establish new habits and lifestyle changes beyond the institutional setting [8–10]. The same requirements also characterize good quality in rehabilitation [11]. However, important gaps persist between these recommendations and current delivery of rehabilitation services [12]. In Norway, measures to improve the quality of rehabilitation have been recommended particularly to address the documented lack of coordination and communication across care levels and the lack of patient involvement in planning of follow-up interventions after rehabilitation [13, 14].

Although “quality” is a rather abstract term, the use of quality indicators (QIs) may enable practical evaluation and improvement of quality [15]. A QI can be defined as “a measurable element of practice performance for which there is evidence or consensus that it can be used to assess the quality, and hence change in the quality, of care provided” ([16], p. 104). QIs often are related to Donabedian’s model of quality in health care and the interplaying triad of structure, process, and outcomes of care [15, 17–20].

An expert group of researchers, patient research partners, and clinicians in Norway has recently developed a set of QIs for monitoring, evaluating, and improving the quality of rehabilitation in RMDs [21]. The QI set consists of two separate questionnaires: one for rehabilitation providers (addressing structure QIs) and one for patients (addressing process and outcome QIs) [21]. Developers and users of the instrument used the Rand/UCLA Appropriateness Method to agree on content validity [21]. In the pilot testing, the QI set was appraised as feasible for monitoring quality in rehabilitation in primary and secondary care, and face validity was regarded as good [21], but further investigation of measurement properties was suggested. Especially, the QI set’s ability to detect change over time (responsiveness) was of interest for its use in measuring quality improvement in rehabilitation services. Thus, the aim of our study was to assess the responsiveness of a quality indicator set for rehabilitation for people with RMDs [21].

## Methods

### Study design and clinical settings

We tested the QI set in the multicenter stepped-wedge, cluster-randomized controlled BRIDGE trial [22], which aimed to improve continuity and quality in rehabilitation for people with RMDs. The National Advisory Unit on Rehabilitation in Rheumatology recruited participating rehabilitation centers (*n* = 8) in different regions of Norway. The centers started the trial simultaneously and acted as controls (delivering traditional rehabilitation programs) until an allocated point in time for each center to switch to the intervention phase (adding the new BRIDGE program to the traditional programs). Assessment of the responsiveness of the QI set was performed as a pre–post evaluation, before and after the addition of the new BRIDGE program.

Health professionals at the centers recruited patients at admission to rehabilitation. Patient-reported data were collected at admission and discharge from rehabilitation in secondary care and in the subsequent follow-up period at home (2, 7, and 12 months after admission). Eligible patients were aged ≥18 years and admitted to rehabilitation with one of the following diagnoses: inflammatory rheumatic diseases, systemic connective tissue diseases, osteoarthritis, osteoporosis, fibromyalgia or widespread pain, or non-specific low back, neck, or shoulder pain (persistent for more than 3 months). Because the electronic data collection and questionnaires were available only in Norwegian, patients needed to be proficient in Norwegian and to have a personal electronic credential for secure identification online. Further, they needed internet connection, and a personal computer, tablet computer, or smartphone. Patients with fracture(s), cognitive impairment, or severe psychiatric disorder(s) were excluded. Eligible patients received verbal and written information about the study. Those who decided to participate provided written informed consent. The study was approved by the Norwegian Regional Committee for Medical Research Ethics (REK South-East, 2017/665).

#### The BRIDGE program

The main elements of the BRIDGE program are described in Table 1. At each center, the providers used a fidelity check list to monitor whether they delivered the program according to the BRIDGE protocol.

**Table 1:** Elements of the BRIDGE program, aimed at strengthening the quality of rehabilitation services

*Structured goal-setting*	Patients developed 1–5 individual rehabilitation goals in collaboration with clinicians. The goals were recorded in the Patient-Specific Functional Scale [23, 24], and scored according to experienced difficulty at every reporting time point in the trial.
*A written rehabilitation plan*	A written rehabilitation plan for each patient included the individual goals and corresponding goal-directed interventions.
*A tailored follow-up, including plans for self-management*	The patient and the rehabilitation team developed a plan for tailored follow-up in the first period after discharge. One month after discharge, all participants received a telephone call from the rehabilitation center, addressing 1) progress towards goals, 2) adherence to self-management strategies (plans for self-management), and 3) whether necessary contact with caregivers in the patient’s home setting was established. The follow-up interventions were tailored according to patient’s needs and available resources in their municipality.
*Individualized written feedback*	Digital self-reporting enabled individualized graphic feedback throughout the whole rehabilitation period. Data reported in a rehabilitation core set of questionnaires were presented as clinical graphs showing current status and development over time. Participants could use the graphs to monitor their own progress and share information with important caregivers across levels of care.
*Motivational interviewing*	Motivational interviewing was used in the goal-setting talks and the telephone follow-up calls, in accordance with guiding booklets designed for both clinicians and patients.

### Data collection and measurements

At two time points, the head of each center completed the center-reported QI questionnaire in telephone-based interviews conducted by the central project coordinator (ALSS). The first interview was performed at the beginning of the study while the centers were still delivering traditional programs (T1). Using an interview guide based on the Scandinavian Team Arthritis Register-European Team Initiative for Care Research (STAR-ETIC) rehabilitation framework [25], the head of each center also gave detailed information about the content and organization of the rehabilitation program delivered at T1. The second interview took place 6–8 weeks after the addition of the BRIDGE program (T2).

Two months after the rehabilitation stay, all patients completed the patient-reported QI questionnaire. We collected patient-reported T1 data from patients participating in traditional rehabilitation programs (the T1-group) and T2 data from patients participating in the BRIDGE program (the T2-group). In this manner, we measured quality of rehabilitation services (at the institutional level) at T1 and T2 from the perspective of the users.

#### A QI set for the rehabilitation of people with RMDs

Providers completed a questionnaire addressing 19 structure indicators of quality. These indicators measured organizational aspects in which the rehabilitation occurs, e.g., whether written procedures, method descriptions, and/or checklists are currently available and part of the daily routine.

Patients responded to another questionnaire, comprising 14 indicators regarding process and outcome indicators of quality. Process indicators (*n* = 11) measure factors related to giving and receiving care, in the form of actions and interactions between providers and patients in the actual clinical setting [20, 21]. Outcome indicators (*n* = 3) measure the effects of rehabilitation on defined outcomes, related to attainment of rehabilitation goals, improvements in function, and/or improvements in health-related quality of life [20, 21]. Taken together, the main themes covered by the QI set are as follows: 1) patient participation in goal setting and the rehabilitation process; 2) follow-up plan and continuity across levels of care; and 3) assessment, outcomes, and time-points of evaluation. The QI set is presented in Table 2. The content of many structure indicators matches the content of process and/or outcome indicators, which allows for measuring quality in rehabilitation services from the system and user perspectives, respectively.

**Table 2:** Main themes and indicators in a quality indicator set for use in rehabilitation [21]

Main themes	Structural quality indicators/center-reported:		Process quality indicators/patient-reported:	
	I	Question (yes/no)	I	Question (yes/no)
Patient participation in goal setting and rehabilitation process	**C01**	C1. P shall participate in setting rehab goals	**P04**	P4. Were you actively involved in setting specific goals for the rehab period?
	**C02**	C2. P shall participate in planning his/her rehab process.		
	**C03**	C3. A template is used to prepare an individual rehab plan for P.	**P03**	P3. Was a written plan developed for the rehab period (comprising your rehab goals, what you should practice, etc.)?
			**P05**	P5. Were you actively involved in preparing a specific written plan for the rehab period (mentioned in q. 3)?
	**C04**	C4. P shall participate in evaluating his/her ongoing process.	**PO6**	P6a. Did you participate in at least two meetings with the team^a^ during which your goal(s) and goal attainment so far were discussed?
	**C05**	C5a. There are at least two meetings between P and the team^a^.		
Follow-up plan and continuity across levels of care	**C09**	C7a. P shall participate in preparing a specified written follow-up plan (aside from the epicrisis) for the follow-up process after the rehab period. This plan shall also include P’s own efforts to maintain or improve function/health.	**P09**	P7. Apart from regular epicrisis, was a written plan developed for the period after rehab, including what you were expected to work on yourself? *(if you have answered “yes” to q. 7, go to q. 8. If you have answered “no” to q. 7, go to q. 9)*
			**P10**	P8a. Did you participate in developing the plan (q. 7)?
	**C10**	C7b. If there is a need for health care support after the rehab period, the relevant personnel are to be informed about the plan or participate in the development of the follow-up plan.	**P11**	P8b. As a part of this plan, were you consulted about whether you needed follow-up from external personnel^b^ after the rehab. Period?
	**C06**	C5b. P is asked before meetings if he/she wants his/her next of kin to attend any of the meetings.	**P07**	P6b. Were you asked if you wanted your next of kin to attend any of the meetings?
	**C07**	C5c. P is asked before meetings if he/she wants some of the external professionals^b^ he/she will relate to after the rehab. to attend any of the meetings.	**P08**	P6c. Were you asked if you wanted professionals^b^ you will relate to after the rehab period to attend any of the meetings?
Assessment, outcomes, and time-point of evaluation	**C08** **C11** **C12** **C13** **C14** **C15** **C16** **C17** **C18** **C19**	C6. The rehab unit uses reliable^c^ questionnaires and/or functional tests to assess physical, mental, and/or social conditions. P’s goal/goal attainment is to be assessed … C8a … .with a reliable^c^ instrument. C8b. … at the beginning and the end of the rehab period. C8c. … 3–6 months after the rehab period. P’s function is to be registered … C9a … using a reliable^c^ instrument. C9b. … at the beginning and the end of the rehab period. C9c. … 3–6 months after the rehab period. P’s health-related quality of life is to be assessed … C10a. … using a reliable^c^ instrument. C10b. … at the beginning and the end of the rehab period. C10c. … 3–6 months after the rehab period.	**P01**	P1. Were your health condition and life situation assessed during the first days of your rehab period? (Answer “no” if both aspects were not assessed) *(If you have answered “yes” to question number 1, go to question number 2. If you have answered “no” to question number 1, go to question number 3).*
			**P02**	P2. Did the assessments include both a physical examination and questions about mental and social conditions, network, home situation, and – if relevant – your work situation?
			**Outcome quality indicators/patient-reported:**	
			**P12**	P9. As a result of the rehab period, have you achieved one or several goals that are important to you?
			**P13**	P10. As a result of the rehab period, have you achieved an improvement in your physical, mental, and/or social functioning that is important to you?
			**P14**	P11. As a result of the rehab period, do you think your quality of life has improved?

*I* Indicator number, *Cx* Center-reported + question number, *Px* Patient-reported + question number, *P* The patient/user, *rehab* Rehabilitation, *q* question number, ^a^the team = the interdisciplinary team, or a professional representing the team; ^b^external professionals = external personnel, such as a physiotherapist, general practitioner, or – if relevant – the labor and welfare administration or a person from patient’s workplace; ^c^reliable = quality-assured/validated questionnaires or tests

**Fig. 1:**
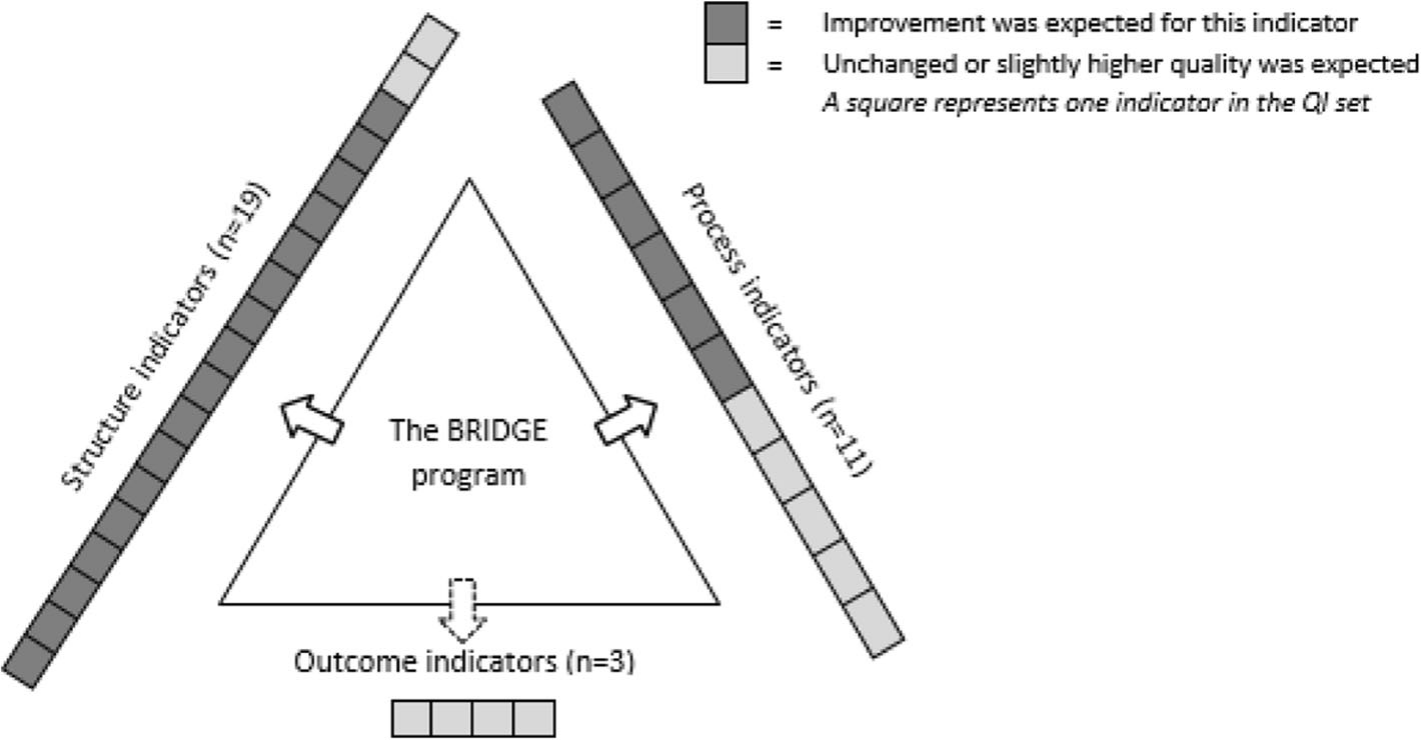
Expected influence of the BRIDGE program on “A quality indicator set of rehabilitation for RMDs”

Because the elements in the BRIDGE program (Table 1) to a large degree mirror the items in the QI set (Table 2), we expected that the QI set would capture improved or maintained quality of rehabilitation between T1 and T2 (Fig. 1). *Maintained* quality was favorable if the quality at T1 already was in line with the normative standards reflected in the quality indicators. If not, *improved* quality was favorable.

##### Response options and scoring alogrithm

Achievements (yes/no) of items in the QI set were measured using pass rates (PRs). Based on responses from the participant (provider or patient), calculations comprised single indicator PRs and total PRs. Single indicator PRs were calculated as the total number of participants who answered “yes” for a particular indicator divided by the total number of participants who answered “yes” or “no” for the same indicator. The scores were normalized to 100 to allow PRs to be reported as percentages.

Single indicator PRs range from 0 to 100% (100% = all eligible participants answered “yes” to this indicator). Total PRs represent the total of “yes” answers from a participant divided by eligible QI items (denominator) for the same participant. Eligible QI items in the center-reported questionnaire are always *n* = 19. Eligible QI items in the patient-reported questionnaire are at least *n* = 11 out of 14 but can vary. As an example: A patient who answers “yes” to question 1 (P1) goes to the additional question 2 (P2) (as seen in Table 2), resulting in n = 11 + 1 for a denominator of 12. In the same way, an answer “yes” to question 7 (P7) makes questions 8a (P8a) and 8b (P8b) eligible, resulting in n = 11 + 2, for a denominator of 13. Finally, “yes” answers to both questions 1 and 7 result in n = 11 + 1 + 2, for the maximum denominator of 14. Total PRs also range from 0 to 100%, with 100% indicating the best quality in rehabilitation score, implying that the participant answered “yes” to all eligible items in the particular questionnaire.

#### The STAR-ETIC rehabilitation framework

The STAR-ETIC framework was developed for describing complex rehabilitation interventions and comparing the content of rehabilitation programs across different sites [25, 26]. We used the framework to collect information about content and organization of the rehabilitation program delivered at T1. The framework covers clinical setting; type of professions in the rehabilitation team; standards for family involvement and follow-up-management; use of rehabilitation goals, assessments, and evaluations; interventions (content and modalities); and outcomes.

##### Other measurements

We obtained demographic data about the patients at baseline. To assess the impact of data clustering from the multicenter design, we also used baseline data for the primary and secondary outcomes in the BRIDGE trial. The primary outcome was goal attainment, as measured by the Patient-Specific Functional Scale (PSFS) [23, 24]. Secondary outcomes were physical function, measured by the 30-s sit-to-stand test (30 secSTS) [27–29], and health-related quality of life (HRQoL), measured by the EuroQoL 5D- 5L-health-related quality of life (EQ. 5D-index and EQ. 5D-vas) [29, 30]). Norwegian versions of all instruments, translated following international guidelines, have been tested for psychometric properties with satisfactory results in RMD populations in rehabilitation settings in primary and secondary care [29].

On the PSFS (open-ended categories), patients report up to five activites that they currently find difficult to perform because of their health condition. Each activity is scored according to experienced performance on an 11-point scale (0–10, with 0 indicating “unable to perform”) [24, 29]. In the EQ. 5D-index, patients report their level of perceived problems in five dimensions of health (mobility, self-care, usual activities, pain/discomfort, and anxiety/depression; 5 levels, with 1 indicating no problems and 5 indicating extreme problems). In the EQ. 5D-vas, patients rate their current health state on a 100-mm visual analog scale (0–100, with 0 indicating “*The worst health you can imagine*” and 100 indicating “*The best health you can imagine*”) [29, 30]. In the performance-based test (30 secSTS), the patient, seated in a chair, rises to a full standing position and then sits down again. According to specific performance instructions, patients complete as many full stands as possible within 30 s [28, 29].

### Responsiveness

Responsiveness has been defined by the COnsensus-based Standards for the selection of health status Measurement INstruments (COSMIN) panel as “the ability of an instrument to detect change over time in the construct to be measured” ([31], p. 742). In this study, we used a construct approach to examine responsiveness [32] because no gold standard is available. Based on current evidence, previous pilot testing [21], and the BRIDGE fidelity checklist, three of the authors (IK, GB, and ALSS) developed a priori hypotheses regarding the expected direction and magnitude of PR changes between T1 and T2. We discussed our hypotheses in a research group with nurses, patient research partners, and a physiotherapist. In accordance with de Vet [33], high responsiveness was indicated if at least 75% of the predefined hypotheses were confirmed.

The rationales for the hypotheses were based on results from the pilot study, other previous research, expert opinions, and fidelity checklist and guiding booklets available in the BRIDGE trial. The rationales are given in detail in Additional file 3*.* In short, we developed four hypotheses for median total PRs and 1–3 hypotheses for PR changes for each single indicator. Regarding total PR changes, we included hypotheses for the largest diagnose groups in our trial (inflammatory rheumatic disease, and fibromyalgia/widespread pain, respectively). We expected the change score for total PR to be small to moderate for both subgroups, applied to process and outcome indicators, respectively. Regarding single indicators, we expected improved PRs for QIs that were addressed by the BRIDGE program: patient participation in 1) setting goals, 2) developing a written rehabilitation plan, 3) meeting(s) where goals and/or ongoing rehabilitation process were discussed, 4) consultation(s) about needs for the follow-up period, 5) developing a written follow-up plan, and 6) involvement of externals in planning follow-up. Concerning assessments and time-points of evaluation, we expected improved PRs for 1) use of reliable questionnaires/tests, 2) evaluation of goal attainment, function, and HRQoL at the start and end of the rehabilitation intervention in specialist care, and 3) 3–6 months after discharge (structure). We expected no change for QIs regarding initial bio-psycho-social assessment (process) and no change or little improvement for QIs regarding patient’s outcomes. Involving externals (i.e., next of kin or services in primary care) was expected as part of the follow-up plan, but invitations to meetings for next of kin or external services were not included in the BRIDGE program. Hence, we did not expect changes in QIs regarding invitation to meetings for next of kin or external services.

### Data analysis

We used STATA IC v14 for statistical analysis*.* To compare the baseline characteristics of patients in the T1- and T2-groups, we used the independent samples t-test, Pearson’s Chi square test, and the Mann–Whitney U test. We set the significance level at 0.05. To assess the impact of clustering in each group, we calculated intraclass correlation coefficients (ICCs) for primary and secondary outcomes.

In testing hypotheses regarding responsiveness, we used descriptive statistics to examine the median PR values and change scores for total PRs and single indicator PRs, respectively.

Based on absolute changes, we used the following criteria for indicating the magnitude of changes: 1) 0%, no change; 2) 1.0–12.5%, small change (change for 1/8 participating centers); 3) 12.6–25%, moderate change (change for 2/8 participating centers); and 4) 25.1–100%, considerable change (change for 3 or more participating centers). We used the same criteria for the magnitude of changes in patient-reported quality: 1.00–12.5%, small change; 12.6–25%, moderate change; and 25.1–100%, considerable change.

Returned QI questionnaires were considered incomplete and not included in further analyses if more than 50% of the QI items had not received a “yes” or “no” response.

## Results

### Rehabilitation at participating centers

All eight centers were organized in secondary care (specialized rehabilitation), with a minimum of four different professions in the multidisciplinary teams. The teams included physicians, physiotherapists, occupational therapists, nurses, and social workers in all centers except center 6 (no nurse) and center 4 (no social worker). Additionally, the teams included a nutritionist or dietitian at six centers, a sport educator at three centers, and a psychologist at one center. Most centers delivered inpatient stays for 3–4 weeks, and two hospital departments delivered a shorter stay (2 weeks), as either inpatient (center 2) or outpatient (center 6) rehabilitation. Length of stay was predetermined, but postponed discharge was allowed in cases of vacancy (centers 1, 3, 4, 8). The rehabilitation programs were developed for different patient groups. The primary group was inflammatory arthritis at all the hospital departments and 2/5 rehabilitation centers, fibromyalgia/widespread pain at centers 4 and 7, and unspecific low back, neck, or shoulder pain at center 5 (see Additional file 1).

At all centers, the content of rehabilitation comprised a combination of group sessions, individual sessions, and self-training. The treatment sessions were comprehensive, including topics like training, physical activity, activities of daily living, pacing, planning and adaptations. Further, counseling regarding coping (pain, fatigue, sleep, or stress), lifestyle changes (physical activity, exercise, weight control, smoking), disease information and medical treatments. Topics like family and other social relationships, work and work adaptations, social services and rights were also included, as well as mindfulness and relaxation.

### Patient participants

The study included 357 participants (200 in the T1-group, 157 in the T2-group), and their characteristics are summarized in Additional file 2. The groups were comparable for all baseline variables except age, diagnosis, and disease duration (Additional file 2). The differences in age and disease duration were not considered clinically important, and except for differences in diagnoses, the between-group comparability was considered acceptable. Most patients had inflammatory rheumatic disease (72% in the T1-group, 54% in the T2-group), or fibromyalgia/widespread pain (10, 32%). For other patients the primary diagnose was unspecific low back-, neck-, or shoulder pain, connective tissue disease, or osteoarthritis. None of the included patients had osteoporosis as the primary diagnose (see Additional file 2).

The patients who did not complete the QI questionnaire did not differ systematically by baseline.

### Assessment of responsiveness

The ICCs for the outcomes of interest were small (ICC_psfs_ = 0.08, ICC_30sec_ = 0.03, ICC_EQ5Dindex_ = 0.06, ICC_EQ5Dvas_ = 0.02), indicating a low impact of clustering. Consequently, we pooled patient-reported data from different centers for calculations of total PRs and single indicator PRs.

A total of 161/200 (80.5%) patients in the T1-group and 132/157 (84%) in the T2-group, completed the QI questionnaire. The response rate from participating centers was 100% (no missing items).

Among 62 predefined hypotheses for change in single indicator PRs, 9 (14.5%) were not applicable because of the observed distribution of answers at T1. For three structure indicators, there were no “yes” answers at T1, so that hypotheses about “*all centers who answered ‘yes’ at T1 are expected to answer ‘yes’ at T*2” were not applicable (*n* = 3). For three other structure indicators, there were zero “no” answers at T1, so that the following hypotheses were not applicable: “*all centers who answered ‘no’ at T1 are expected to answer ‘yes’ at T2*” (n = 3 hypotheses), and “*the change score for this indicator is expected to be [magnitude of change is described]*” (*n* = 3).

Of the remaining 53 hypotheses for single indicators, 44 (83%) were confirmed. Regarding change scores in median total PRs, three of four hypotheses were confirmed. Taken together, the observed change scores were consistent with ≥75% of the predefined hypotheses, indicating adequate responsiveness for the rehabilitation QI set. These findings are presented in more detail in Table 3 and Additional file 3.

**Table 3:** Expected and observed change scores for quality indicators

**a. Changes in median total pass rates**					
	**Hypo-theses**	**Confirmed direction**^**1**^ **of change**	**Expected magnitude of change**	**Observed magnitude of change**	**Confirmed hypothesis**
**Structural QIs** (center-reported, *n* = 8)	H_total_ 1	yes	moderate to high	high	1/1
**Process and outcome QIs** (patient-reported, *n* = 132–161)	H_total_ 2	yes	small to moderate	small	1/1
**Process QIs in subgroups** (subgroup1 = inflammatory rheumatic disease, *n* = 74–114) (subgroup2 = fibromyalgia or chronic widespread pain, *n* = 14–40)	H_total_ 3	yes	small to moderate (both groups)	small (both groups)	1/1
**Outcome QIs in subgroups** (subgroup1 = inflammatory rheumatic disease, *n* = 74–114) (subgroup2 = fibromyalgia or chronic widespread pain, *n* = 14–40)	H_total_ 4	yes	zero to small	zero (subgroup1) moderate (subgroup2)	0/1
**IN TOTAL (changes in median total pass rates)**					**3/4 confirmed**
**b. Changes in single items pass rates**					
**Structural QIs (center-reported, marked C)** **Process QIs (patient-reported, marked P)**	**Hypo-theses**	**Confirmed direction**^**1**^ **of change**	**Expected magnitude of change**	**Observed magnitude of change**	**Confirmed hypothesis**
*Patient participation in goal setting and rehabilitation process*					
**C01**. P shall participate in setting rehab goals.	H_single_ 1	yes	All (100%)	All (100%)	
	H_single_ 2	yes	small to moderate	moderate	2/2
**P04**. Were you actively involved in setting goals for the rehab period?	H_single_ 3	Yes	Similar or small	small	1/1
**C02**. P shall participate in planning his/her own rehab process.	H_single_ 4	yes	All (100%)	All (100%)	
	H_single_ 6	yes	small to moderate	moderate	2/2
**C03**. A template is used to prepare an individual rehab plan for P.	H_single_ 5	yes	All (100%)	All (100%)	
	H_single_ 7	yes	small to moderate	high	1/2
**P03**. Was a written plan developed for the rehab period (comprising your rehab goals, what you should practice, etc.)?	H_single_ 8	Yes	moderate	small	0/1
**P05**. Were you actively involved in preparing the written rehab plan?	H_single_ 9	Yes	moderate	small	0/1
**C04**. P shall participate in evaluating his/her ongoing process.	H_single_ 13	yes	All (100%)	All (100%)	
	H_single_ 14	yes	small to moderate	high	1/2
**C05**. There are at least two meetings between P and the team^a^.	H_single_ 10	yes	All (100%)	All (100%)	
	H_single_ 11	yes	small to moderate	small	2/2
**P06**. Did you participate in at least two meetings with the team^a^ at which your goal(s) and goal attainment so far were discussed?	H_single_ 12	Yes	moderate	small	0/1
*Follow-up plan and continuity across levels of care*					
**C09**. P shall participate in preparing a specific written follow-up plan (aside from the epicrisis) for the follow-up process after the rehab period. This plan shall also include P’s own efforts to maintain or improve function/health.	H_single_ 15	yes	All (100%)	All (100%)	
	H_single_ 16	yes	small to moderate	high	1/2
**C10**. If there is a need for health care support after the rehab period, the relevant personnel are to be informed about the plan or participate in the development of the follow-up plan.	H_single_ 17	yes	All (100%)	All (100%)	
	H_single_ 18	yes	moderate to high	high	2/2
**P09**. Was a written plan developed for the period after rehab, including what you were expected to work on yourself?	H_single_ 19	Yes	small to moderate	moderate	1/1
**P10**. (if “yes” to q. 7): Did you participate in developing the plan (in q. 7)?	H_single_ 20	Yes	small to moderate	small	1/1
**P11**. As part of this plan, were you consulted about whether you needed follow-up from external personnel after the rehab period?	H_single_ 21	Yes	small to moderate	moderate	1/1
**C06**. P is asked before meetings if he/she wants his/her next of kin to attend any of the meetings.	H_single_ 22	yes	All (100%)	All (100%)	
	H_single_ 23	yes	zero to small	zero	2/2
**P07**. Were you asked if you wanted your next of kin to attend any of the meetings?	H_single_ 24	Yes	zero to small	small	1/1
**C07**. P is asked before meetings if he/she wants some of the professionals^b^ he/she will relate to after the rehab to attend any of the meetings.	H_single_ 25	n.a.	All (100%)	n.a.	n.a.
	H_single_ 26	yes	zero to small	small	1/1
**P08**. Were you asked if you wanted external personnel^b^ to attend any of the meetings?	H_single_ 27	Yes	zero to small	small	1/1
**Structural QIs (center-reported, marked C)** **Process QIs (patient-reported, marked P)**	**Hypo-theses**	**Confirmed direction**^**1**^ **of change**	**Expected magnitude of change**	**Observed magnitude of change**	**Confirmed** **hypothesis**
*Assessment, outcomes, and time-points of evaluation*					
**P01**. Were your health condition and life situation assessed during the first days of your rehab period?	H_single_ 28	Yes	zero to small	small	1/1
**P02**. (if “yes” to q. 1): Did the assessments (in q. 1) include both a physical examination, and q.about mental, and social conditions, network, home situation and – if relevant – your work situation?	H_single_ 29	Yes	zero to small	small	1/1
**C08**. The rehab unit uses reliable^c^ questionnaires and/or functional tests to assess physical, mental, and/or social conditions.	H_single_ 30	yes	All (100%)	All (100%)	1/1
	H_single_ 34	n.a.	All (100%)	n.a.	n.a.
	H_single_ 38	n.a.	small to moderate	n.a.	n.a.
P’s goal/goal attainment is to be assessed …					
**C11**. … with a reliable instrument	H_single_ 31	yes	All (100%)	All (100%)	
	H_single_ 35	yes	All (100%)	All (100%)	
	H_single_ 39	yes	moderate to high	high	3/3
**C12**. … at the beginning and the end of the rehab period	H_single_ 42	yes	All (100%)	All (100%)	
	H_single_ 45	yes	All (100%)	All (100%)	
	H_single_ 48	yes	moderate to high	small	2/3
**C13**. … 3–6 months after the rehab period	H_single_ 51	yes	All (100%)	All (100%)	
	H_single_ 54	yes	All (100%)	All (100%)	
	H_single_ 57	yes	moderate to high	high	3/3
P’s function is to be registered …					
**C14**. … using a reliable instrument	H_single_ 32	yes	All (100%)	All (100%)	1/1
	H_single_ 36	n.a.	All (100%)	n.a.	n.a.
	H_single_ 40	n.a.	small	n.a.	n.a.
**C15**. … at the beginning and the end of the rehab period	H_single_ 43	yes	All (100%)	All (100%)	1/1
		n.a.			
	H_single_ 46	n.a.	All (100%)	n.a.	n.a.
	H_single_ 49		small	n.a.	n.a.
**C16**. … 3–6 months after the rehab period	H_single_ 52	n.a.	All (100%)	n.a.	n.a.
	H_single_ 55	yes	All (100%)	All (100%)	
	H_single_ 58	yes	moderate to	high	2/2
P’s health-related quality of life is to be assessed …					
**C17**. … using a reliable instrument	H_single_ 33	yes	All (100%)	All (100%)	
	H_single_ 37	yes	All (100%)	All (100%)	3/3
	H_single_ 41	yes	moderate to high	high	
**C18**. … at the beginning and the end of the rehab period	H_single_ 44	yes	All (100%)	All (100%)	
	H_single_ 47	yes	All (100%)	All (100%)	
	H_single_ 50	yes	moderate to high	high	3/3
**C19**. … 3–6 months after the rehabperiod	H_single_ 53	n.a.	All (100%)	n.a.	n.a.
	H_single_ 56	yes	All (100%)	All (100%)	
	H_single_ 59	yes	moderate to high	high	2/2
As a result of the rehab					
**P12**. … have you achieved one or several goals that are important to you?	H_single_ 60	No	zero to small	small	0/1
**P13**. … have you achieved an improvement in your physical, mental, and/or social functioning that is important to you?	H_single_ 61	Yes	zero to small	small	1/1
**P14**. … do you think your quality of life has improved?	H_single_ 62	No	zero to small	small	0/1
**IN TOTAL (changes in single item scores)**					**44/53 confirmed**

*1* expected direction is positive or stable for all the hypotheses , *QI* quality indicator, *Htotalxx* hypotheses concerning change in total pass rates, followed by hypothesis number,  *Hsinglexx* hypotheses concerning change in single indicator pass rates, followed by hypothesis number, *rehab* rehabilitation, *q* question; ^a^the team = the interdisciplinary team or a professional representing the team; ^b^external personnel, such as a physiotherapist, general practitioner, or – if relevant – the labor and welfare administration or a person from work; ^c^quality-assured/validated questionnaires or tests, *n.a.* Not applicable

#### Direction of change

**Fig. 2:**
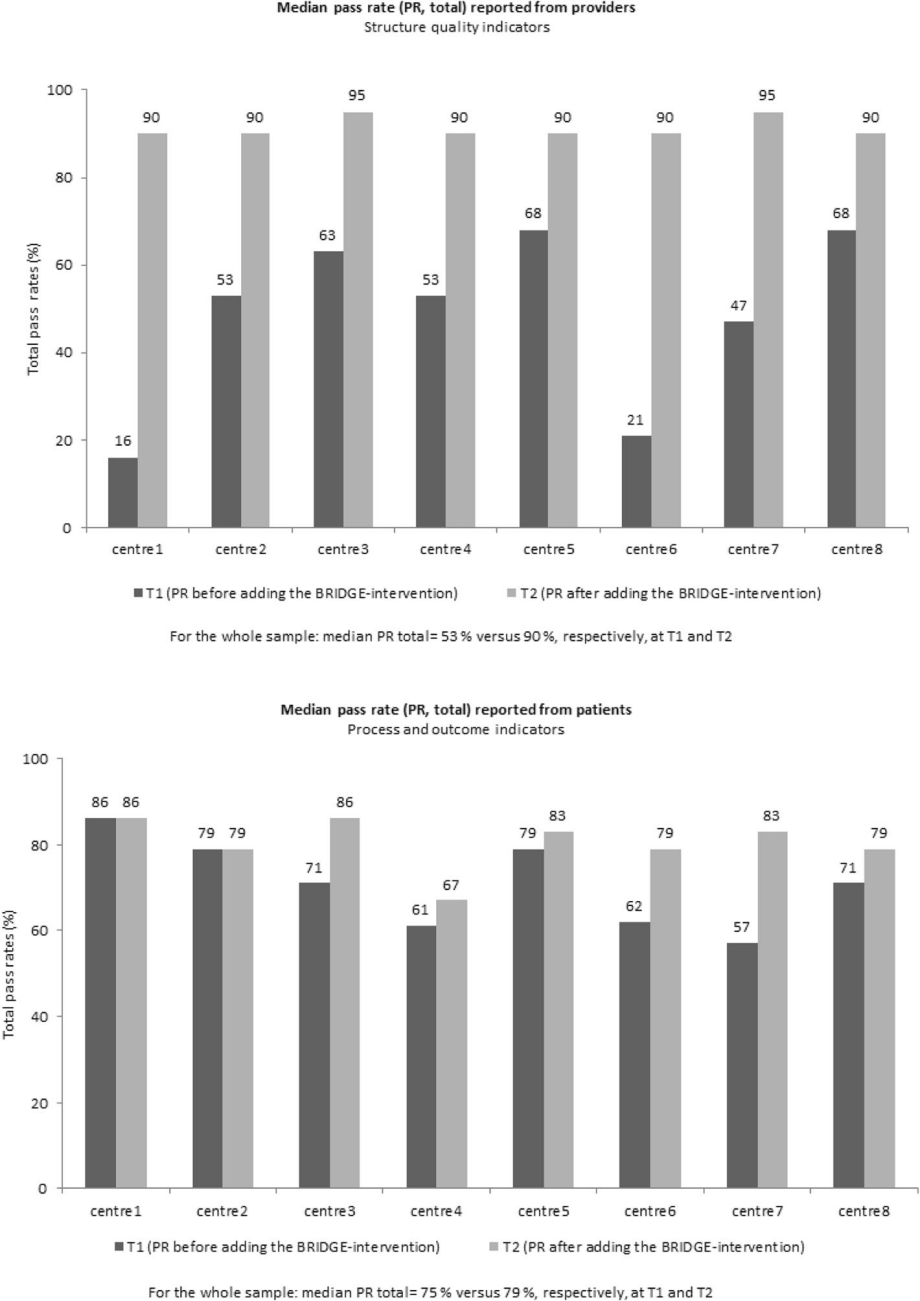
Longitudinal changes in total pass rates in the time interval from T1 to T2

As hypothesized, the changes in total PRs were in the direction of improvement for all dimensions of quality in rehabilitation (structure, process, and outcomes), with the largest improvements for structure indicators. The center-reported quality at T2 was high and comparable across all participating centers (PR total ranging from 90 to 95%), in spite of differences at T1 (PR total ranging from 16 to 68%) (Fig. 2). All but two hypotheses for single indicators were also confirmed. However, there was a negative direction for two out of three hypotheses concerning outcomes, for which a positive was expected: H_single_60 (achieved important goals) and H_single_62 (improved quality of life; see Table 3).

#### Magnitude of change

The expected magnitudes of change were confirmed for each structure indicator, with four exceptions (Table 3): observed improvement was smaller than expected for C12 (patient’s goal/goal attainment is to be assessed with a reliable instrument at the beginning and the end of the rehabilitation period), and observed improvements were larger than expected for C03 (use of a template to prepare a rehabilitation plan for the patient), C04 (patient participation in evaluation of their ongoing process), and C09 (patient participation in preparing a written follow-up plan), respectively. In contrast to the results for C03, the observed improvements were smaller than expected for the matching process indicators P03 and P05 (patient participation in developing and use of a written rehabilitation plan). Smaller improvement than expected was also found for the process indicator P06 (participating in at least two meetings with team member(s)).

**Fig. 3:**
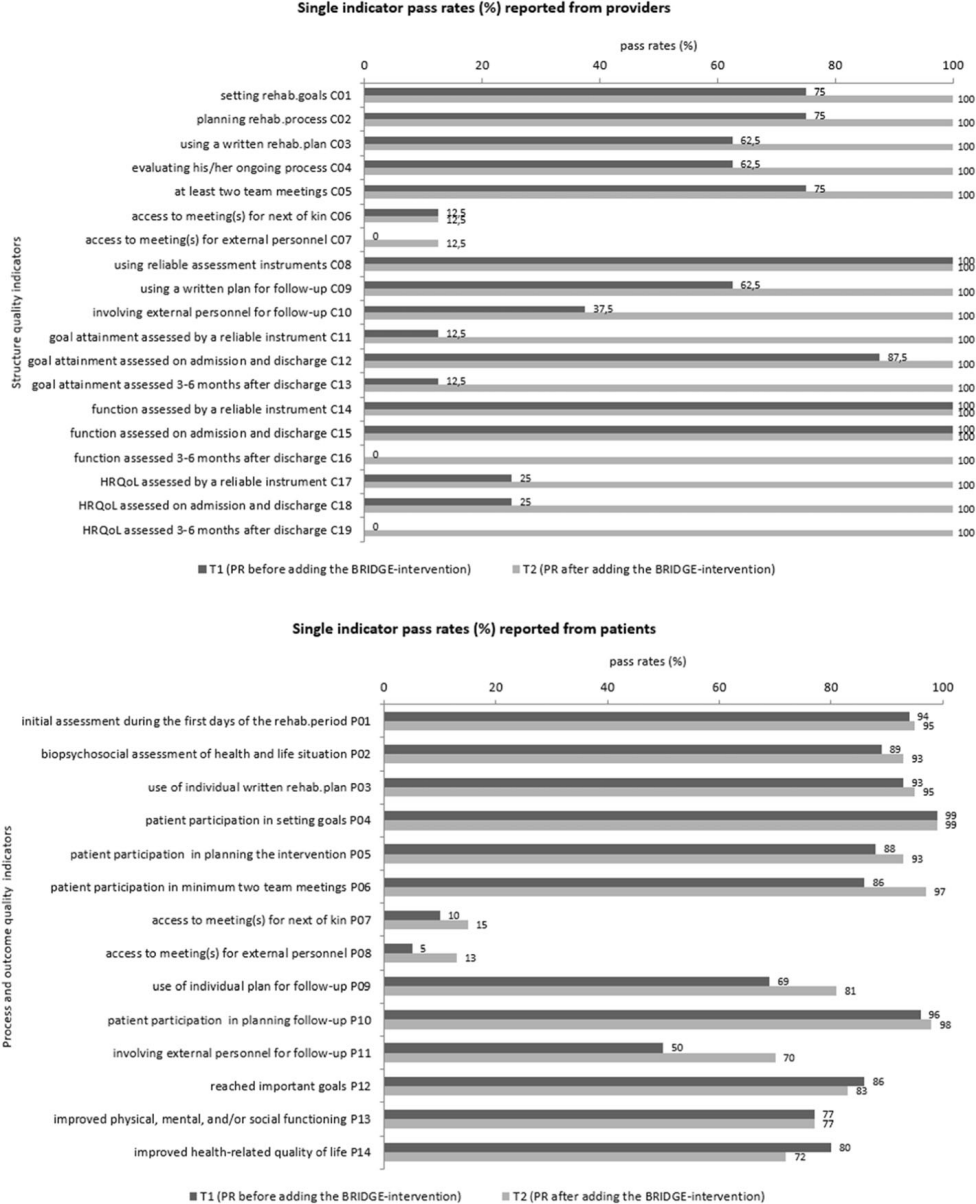
Longitudinal changes in single indicator pass rates in the time interval from T1 to T2

As hypothesized, PRs were particularly low for indicators concerning access to meetings for next of kin or external personnel at T1 and T2, respectively (Fig. 3). At both points in time, PR values below 16% were observed for both process indicators (P07, P08) and the matching structure indicators (C06, C07) (Fig. 3).

From both the service and the user perspectives, the largest improvements from T1 to T2 were related to externals involved in planning the follow-up (Fig. 3). The change scores were 62.5% for the structure indicator (C10) and 20% for the matching process indicator (P11) (Additional file 3). The magnitude of these improvements confirmed the predefined expectation (Table 3, Additional file 3).

## Discussion

In this study, we evaluated the responsiveness of a newly developed QI set for rehabilitation services for people with RMDs. A construct approach was used, with predefined hypotheses regarding expected changes in QI pass rates after the addition of a new rehabilitation intervention to the traditional programs delivered at eight rehabilitation centers in specialist care. The results show adequate responsiveness, with more than 75% of the predefined hypotheses being confirmed.

Although most of the hypotheses were confirmed, some reasons for unconfirmed hypotheses are worth noting. First, the change scores were larger than expected for three of the structure indicators. When developing the hypotheses, we assumed that implementation of written procedures, which is required for a shift from “no” to “yes” on structure indicators, would be difficult to achieve for the centers. However, more respondents answered “yes” at T2 than expected. One reason may be that providers regarded the BRIDGE booklets for patients and providers as written procedures. Whether the centers continued to use these booklets after the research period would be interesting to explore in a follow-up study.

Second, the change scores were smaller than expected for three of the process indicators, likely because quality was already in line with normative standards at T1. Indeed, we found surprisingly high PR values for the three indicators at T1 (93, 88, and 86%, respectively), and the potential for change in these indicators was therefore negligible. For other indicators, we had several hypotheses (*n* = 16) regarding maintenance of good quality from T1 to T2, which were confirmed. Consequently, our data suggest that the QI set will capture efforts to improve or prove good quality over time, implying the double intention when monitoring quality: In addition to measuring quality improvements, it is important to know whether established good quality is maintained.

Third, we expected stable or improved outcome indicators both after the traditional rehabilitation program (T1) and after the BRIDGE intervention (T2). In line with these expectations, we found that PR values at T1 and T2 were equal for P13 (improved physical mental, and/or social functioning), whereas the change scores for P12 (reached important goals) and P14 (improved HRQoL) were slightly negative (− 3% and − 8%, respectively). Also, when considering the outcome indicators for one of the subgroups, the observed change score for total PR differed from what we hypothesized. Many factors may have influenced these results, such as variation in patient groups among centers, and factors not captured by the chosen baseline characteristics, such as motivation, ability to be compliant, and individual decisions about when to focus on different goals and issues through the follow-up period. As others have highlighted [19, 20, 34–36], structure and processes of provided care explain only a portion of what influences outcomes. Nevertheless, patient-reported clinical outcomes should remain relevant for monitoring quality because of the expected interplay among all dimensions in the concept of quality [15, 34–37]. However, further research is needed regarding the kind of outcomes that are most sensitive to detecting differences in quality of care and the evidence for potential links among structure, process, and outcome indicators [15, 20, 35–37].

### Strengths and limitations

The strengths of this study include a methodology guided by the COSMIN checklist [32], a large patient sample size, and high data quality with a response rate of 100% for center-reported QIs and more than 80% for patient-reported QIs. However, the use of questionnaires in Norwegian may have induced a sample bias of having few participants from ethnic minority groups. Apart from this, we believe that the study group was representative and that the results may apply to the broad RMD population receiving specialized rehabilitation in Norway [38]. The most important limitation in our study is the modest number of rehabilitation units. However, this manageable sample enabled us to offer tailored guidance to prepare for high fidelity when adding the new BRIDGE program at each center. Moreover, the number of Norwegian institutions in specialized care delivering rehabilitation services for people with RMDs is limited, and our sample include both rehabilitation institutions and hospital rehabilitation departments across rural and urban regions. Still, the indicator set might function differently within rehabilitation services and funding systems abroad. Therefore, responsiveness should be further tested in studies in different countries and levels of care.

Finally, in our evaluation of responsiveness, all hypotheses counted equally. This choice can be questioned because we did not form the same number of hypotheses for each indicator. The greater number of hypotheses for the structure indicators may have led to an unbalanced evaluation of the interplaying triad of structure, process, and outcome indicators. However, we note that we assessed responsiveness for the QI set in its entirety and not for separate subscales. Although center- and patient-reported QIs are separate questionnaires, we recommend that they be used simultaneously to cover the concept of quality from both the service and patient perspectives.

### Implications

Quality of care is receiving increasing emphasis and interest in research, clinical practice, and public documents [12–15]. For different stakeholders, such as patients, health professionals, researchers, and policy makers, it is important to have tools for delivering and demanding optimal rehabilitation [39]. This QI set offers a timely opportunity to establish quality-of-rehabilitation benchmarks, promote important steps toward high-quality rehabilitation, and track trends over time. As far as we know, this QI set is the first indicator set developed for use in rehabilitation for people with RMDs, covering structure, process, and outcome quality [21]. In the pilot study, the QI set was proven feasible, with satisfactory face and content validity [21]. Our results support that the QI set also can be used in longitudinal evaluations of quality in or between rehabilitation services. Such information may be useful for providers in evaluating local quality improvement initiatives or continuing efforts to keep the service in line with the recommendations. Additionally, the information may be useful for policy makers, funders, and researchers in following trends over time and trajectories across care levels and identifying potential problems or issues to consider when planning for future management of rehabilitation. A further important application is the facilitation of patients’ choice of providers, by producing information about the quality of rehabilitation available.

## Conclusion

We found that this QI set for rehabilitation was responsive when applied in rehabilitation services for adults with various RMD conditions. The QI set holds potential as an important tool for capturing changes or monitoring maintenance in the multidimensional arena of quality in rehabilitation. Our results support the use of this QI set in clinical practice and research when the intention is to evaluate quality over time from both the system and user perspectives. This QI set may be useful for quality improvement and benchmarking in and between rehabilitation services.

## Supplementary Information

**Additional file 1.** Organization of rehabilitation programs at participating centers.

**Additional file 2.** Baseline characteristics of patients in the BRIDGE trial when the QI set was distributed.

**Additional file 3.** Predefined hypotheses with rationale and results.

## Data Availability

The datasets used and analysed during the current study are available from the corresponding author on reasonable request.

## Abbreviations

QI: Quality indicator
RMD: Rheumatic and musculoskeletal disease
STAR-ETIC: Scandinavian team-arthritis register – european team initiative for care research
PR: Pass rate
PSFS: Patient-specific functional scale
30secSTS: The 30-s sit-to-stand test
EQ-5D: EuroQoL 5D-5L-health related quality of life
COSMIN: Consensus-based standards for the selection of health measurement instruments
ICC: Intraclass correlation coefficient
PT: Physiotherapist
OT: Occupational therapist
