# Supplementary material for: A quality indicator set for rehabilitation services for people with rheumatic and musculoskeletal diseases demonstrates adequate responsiveness in a pre–post evaluation
Source: BMC Health Serv Res. 2021 Feb 20;21:164. doi: 10.1186/s12913-021-06164-2 (PMC7896401; doi:10.1186/s12913-021-06164-2)
Supplement: Supplementary file 1 — Additional file 1. Organization of rehabilitation programs at participating centers. [file 12913_2021_6164_MOESM1_ESM.docx]

# Additional file 1, in the manuscript:

# A quality indicator set for rehabilitation services for people with rheumatic and musculoskeletal diseases demonstrates adequate responsiveness in a pre–post evaluation

Anne-Lene Sand-Svartrud^1*^, Gunnhild Berdal^1^, Maryam Azimi^2^, Ingvild Bø^3^, Turid Nygaard Dager^1^, Siv Grødal Eppeland^4^, Guro Ohldieck Fredheim^5,^ Anne Sirnes Hagland^6^, Åse Klokkeide^7^, Anita Dyb Linge^8^, Kjetil Tennebø^9^, Helene Lindtvedt Valaas^5^, Ann Margret Aasvold^10^, Hanne Dagfinrud^1^ and Ingvild Kjeken^1^

^1^National Advisory Unit on Rehabilitation in Rheumatology, Diakonhjemmet Hospital, PO Box 23 Vinderen, N- 0319 Oslo, Norway.

^2^Diakonhjemmet Hospital, PO Box 23 Vinderen, N- 0319 Oslo, Norway.

^3^Hospital for Rheumatic Diseases Lillehammer, Margrethe Grundtvigs veg 6, N-2609 Lillehammer, Norway.

^4^Sørlandet Hospital Arendal, PO Box 416 Lundsiden, N-4604 Kristiansand,Norway.

^5^Vikersund Rehabilitation Center, Haaviks vei 25, N-3370 Vikersund, Norway.

^6^Hospital for Rheumatic Diseases Haugesund, PO Box 2175, N-5504 Haugesund, Norway.

^7^Rehabilitering Vest Rehabilitation Center, PO Box 2175, N-5504 Haugesund, Norway.

^8^Muritunet Rehabilitation Center, Grandedata 58, N-6210 Valldal, Norway.

^9^Valnesfjord Health Sports Center, Østerkløftveien 249, N-8215 Valnesfjord, Norway.

^10^Meråker Rehabilitation Center, Østigardsveien 24, N-7530 Meråker, Norway.

*Corresponding author. Correspondence: [anne-lene.svartrud@diakonsyk.no](mailto:anne-lene.svartrud@diakonsyk.no)

| **Additional file 1** Organization of rehabilitation programs at participating centres | | | | | | | | | |
| --- | --- | --- | --- | --- | --- | --- | --- | --- | --- |
| Participating centres | | 1 | 2 | 3 | 4 | 5 | 6 | 7 | 8 |
| **Organization (the same for both T1 and T2):** | | | | | | | | | |
| Healthcare system | Norwegian public healthcare system with equal access to all health care services | x | x | x | x | x | x | x | x |
| Level of care | Secondary level (specialist health care) | x | x | x | x | x | x | x | x |
| Type of setting | Hospital department of rheumatology | x | x |  |  |  | x |  |  |
|  | Specialized rehabilitation institution |  |  | x | x | x |  | x | x |
| Primary  diagnoses  (1=the biggest group, 5=the smallest) | Inflammatory arthritis | 1 | 1 | 1 | - | 2 | 1 | 2 | 1 |
|  | Connective tissue diseases | 2 | 2 | - | - | 4 | 2 | - | 2 |
|  | Wide spread pain or fibromyalgia | - | - | 2 | 1 | 3 | - | 1 | 3 |
|  | Unspecific low back pain, neck- or shoulder pain (persistent>3 months) | - | - | 3 | 2 | 1 | - | 2 | - |
|  | Osteoarthritis | - | - | 2 | - | 5 | - | 3 | 3 |
|  | Osteoporosis | - | - | - | - | - | - | - | - |
| Length of stay | 2 weeks |  | x |  |  |  | x |  |  |
|  | 3-4 weeks | x |  | x | x | x |  | x | x |
| Professions in the rehabilitation team | Medical doctor* | x | x | x | x | x | x | x | x |
|  | Physiotherapist | x | x | x | x | x | x | x | x |
|  | Occupational therapist | x | x | x | x | x | x | x | x |
|  | Nurse | x | x | x | x | x | - | x | x |
|  | Social worker | x | x | x | - | x | x | x | x |
|  | Psychologist | - | x | - | - | - | - | - | - |
|  | Nutritionist or dietist | - | x | x | x | x | - | x | x |
|  | Other | x | x | x | x | x | x | x | x |
| **Further information about organization at T1 (before adding the BRIDGE program):** | | | | | | | | | |
| Communication form in the rehabilitation team | A single team meeting during the stay (the whole team) |  |  |  |  |  | x |  |  |
|  | Weekly team meetings (once a week or more frequent) | x | x | x | x | x | - | x | x |
|  | Other meetings when needed (formal or informal) | x | x | x | x | x | x | x | x |
| Patient participation (PP) | PP in team meetings at admission and discharge | x | x | - | - | - | - | - | - |
|  | PP in all team meetings (the whole team) | - | x | - | - | - | - | - | - |
|  | PP in regular meetings with a representative of the team | x | x | - | x | x | - | - | x |
|  | Group-based PP in team meetings | - | - | - | - | - | - | x | - |
| Family involvement | Standard for family involvement | - | - | x | - | - | - | - | - |
|  | Family involvement based on indication | x | - | x | - | - | x | x | x |
| Follow-up | Standard for follow-up management | - | x | x | - | x | x | x | - |
| Goals | Individual goals defined together with team member(s) | x | x | x | x | x | x | x | x |
|  | Standardized assessment of individual goals | - | - | - | - | x | - | - | - |
| Standardized assessment | On admission | x | x | x | x | x | x | x | x |
|  | At discharge (evaluation) | x | x | x | x | x | x | - | x |
| Treatment by health professionals | On individual levels | x | x | x | x | x | x | x | x |
|  | In group sessions | x | x | x | x | x | x | x | x |
| Self-training | Gym, weights-lifting, swimming or outdoor training | x | x | x | x | x | x | x | x |
| Outcomes | Body function | x | x | x | x | x | x | - | x |
|  | Activity | x | x | x | x | x | x | x | x |
|  | Participation | - | x | - | - | x | - | - | - |
|  | Health-related quality of life | - | - | - | x | - | - | - | x |
|  | Goal attainment | - | - | - | - | x | - | - | - |
|  | Patient satisfaction | x | - | x | - | x | - | - | - |
| *T1/T2=first/second time point in evaluation of responsiveness, x=present (provided), -=not present (not provided), *rheumatologist or specialist in physical medicine and rehabilitation.* | | | | | | | | | |
