# Supplementary material for: A quality indicator set for rehabilitation services for people with rheumatic and musculoskeletal diseases demonstrates adequate responsiveness in a pre–post evaluation
Source: BMC Health Serv Res. 2021 Feb 20;21:164. doi: 10.1186/s12913-021-06164-2 (PMC7896401; doi:10.1186/s12913-021-06164-2)
Supplement: Supplementary file 2 — Additional file 2. Baseline characteristics of patients in the BRIDGE trial when the QI set was distributed. [file 12913_2021_6164_MOESM2_ESM.docx]

# Additional file 2, in the manuscript:

# A quality indicator set for rehabilitation services for people with rheumatic and musculoskeletal diseases demonstrates adequate responsiveness in a pre–post evaluation

Anne-Lene Sand-Svartrud^1*^, Gunnhild Berdal^1^, Maryam Azimi^2^, Ingvild Bø^3^, Turid Nygaard Dager^1^, Siv Grødal Eppeland^4^, Guro Ohldieck Fredheim^5,^ Anne Sirnes Hagland^6^, Åse Klokkeide^7^, Anita Dyb Linge^8^, Kjetil Tennebø^9^, Helene Lindtvedt Valaas^5^, Ann Margret Aasvold^10^, Hanne Dagfinrud^1^ and Ingvild Kjeken^1^

^1^National Advisory Unit on Rehabilitation in Rheumatology, Diakonhjemmet Hospital, PO Box 23 Vinderen, N- 0319 Oslo, Norway.

^2^Diakonhjemmet Hospital, PO Box 23 Vinderen, N- 0319 Oslo, Norway.

^3^Hospital for Rheumatic Diseases Lillehammer, Margrethe Grundtvigs veg 6, N-2609 Lillehammer, Norway.

^4^Sørlandet Hospital Arendal, PO Box 416 Lundsiden, N-4604 Kristiansand,Norway.

^5^Vikersund Rehabilitation Center, Haaviks vei 25, N-3370 Vikersund, Norway.

^6^Hospital for Rheumatic Diseases Haugesund, PO Box 2175, N-5504 Haugesund, Norway.

^7^Rehabilitering Vest Rehabilitation Center, PO Box 2175, N-5504 Haugesund, Norway.

^8^Muritunet Rehabilitation Center, Grandedata 58, N-6210 Valldal, Norway.

^9^Valnesfjord Health Sports Center, Østerkløftveien 249, N-8215 Valnesfjord, Norway.

^10^Meråker Rehabilitation Center, Østigardsveien 24, N-7530 Meråker, Norway.

*Corresponding author. Correspondence: [anne-lene.svartrud@diakonsyk.no](mailto:anne-lene.svartrud@diakonsyk.no)

| **Additional file 2** Baseline characteristics of patients in the BRIDGE-trial when the QI-set was distributed (n=357) | | | |
| --- | --- | --- | --- |
|  | T1-group  (n = 200) | T2-group  (n = 157) | p-value |
| Age, years, mean (min, max) | 52 (21,81) | 49 (18,77) | 0.005^1^ |
| Gender, female, n (%) | 148 (74) | 123(78) | 0.341^2^ |
| Diagnosis, n (%)  Inflammatory rheumatic disease (SpA, PsA, RA, JRA)  Osteoarthritis   Connective tissue disease (SLE, SS, PMR, MCTD)  Fibromyalgia syndrome, CWP  Unspecific neck-, shoulder- and low back pain (>3 months)  Osteoporosis | 143 (72)  8 (4)  14 (7)  20 (10)  15 (8)  0 | 85 (54)  5 (3)  6 (4)  51 (32)  10 (6)  0 | <0.001^2^ |
| Disease duration, years, median (min, max) | 17 (1,67) | 13 (0,68) | 0.014^3^ |
| Comorbidities, n, median (min , max) | 2.5 (0,9) | 3 (0,9) | 0.334^3^ |
| Medication usage  NSAIDs, n (%)  Disease modifying anti-rheumatic drugs (DMARDs), n (%)  TNF-inhibitors, Biosimilars, JAK-inhibitors n (%)  Analgesics, n (%)  Other drugs, n (%) | 80 (43)  68 (37)  42 (23)  131 (70)  135 (73) | 76 (53)  51 (36)  26 (18)  103 (72)  107 (75) | 0.068^2^  0.867^2^  0.329^2^ 0.751^2^ 0.647^2^ |
| BMI (kg/m^2^), median (min, max) | 28 (17,66) | 28 (17,50) | 0.662^3^ |
| Smokers, n (%) | 57 (29) | 37 (24) | 0.330^2^ |
| Snuff users, n (%) | 19 (10) | 13 (9) | 0.704^2^ |
| Education > 12 years, n (%) | 80 (40) | 67 (44) | 0.558^2^ |
| Paid work, n (%) | 85 (43) | 69 (45) | 0.664^2^ |
| Recipients of social security benefits, n (%) | 139 (81) | 120 (87) | 0.178^2^ |
| Living with partner, n (%) | 140 (70) | 103 (67) | 0.485^2^ |
| Physical exercise ≥ 1 per week, n (%) | 123 (62) | 81 (53) | 0.082^2^ |
| General activity ≥ 1 per week, n (%) | 147 (74) | 104 (68) | 0.220^2^ |

*^1^Independent Samples T-test, ^2^Pearson Chi Square test, ^3^Mann Whitney U test. SpA: spondyloarthritis, PsA: psoriatic arthritis, RA: rheumatoid arthritis, JRA: juvenile rheumatoid arthritis, SLE: systemic lupus erythematosus, SS: Sjögren syndrome, PMR: polymyalgia rheumatica, MCTD: mixed connective tissue disease, CWP: chronic widespread pain. Disease duration (symptom debut) and comorbidities are self-reported. NSAIDs: nonsteroidal anti-inflammatory drugs, DMARDS include corticosteroids, TNF: tumor necrosis factor, JAK: Janus Kinase. BMI: body mass index (bodyweight/height^2^). Physical exercise: increased heart rate and breathing for 30 minutes or longer. General activity: social or cultural activities, hobbies, work.*
