# Supplementary material for: A quality indicator set for rehabilitation services for people with rheumatic and musculoskeletal diseases demonstrates adequate responsiveness in a pre–post evaluation
Source: BMC Health Serv Res. 2021 Feb 20;21:164. doi: 10.1186/s12913-021-06164-2 (PMC7896401; doi:10.1186/s12913-021-06164-2)
Supplement: Supplementary file 3 — Additional file 3. Predefined hypotheses with rationale and results. [file 12913_2021_6164_MOESM3_ESM.docx]

# Additional file 3, in the manuscript:

# A quality indicator set for rehabilitation services for people with rheumatic and musculoskeletal diseases demonstrates adequate responsiveness in a pre–post evaluation

Anne-Lene Sand-Svartrud^1*^, Gunnhild Berdal^1^, Maryam Azimi^2^, Ingvild Bø^3^, Turid Nygaard Dager^1^, Siv Grødal Eppeland^4^, Guro Ohldieck Fredheim^5,^ Anne Sirnes Hagland^6^, Åse Klokkeide^7^, Anita Dyb Linge^8^, Kjetil Tennebø^9^, Helene Lindtvedt Valaas^5^, Ann Margret Aasvold^10^, Hanne Dagfinrud^1^ and Ingvild Kjeken^1^

^1^National Advisory Unit on Rehabilitation in Rheumatology, Diakonhjemmet Hospital, PO Box 23 Vinderen, N- 0319 Oslo, Norway.

^2^Diakonhjemmet Hospital, PO Box 23 Vinderen, N- 0319 Oslo, Norway.

^3^Hospital for Rheumatic Diseases Lillehammer, Margrethe Grundtvigs veg 6, N-2609 Lillehammer, Norway.

^4^Sørlandet Hospital Arendal, PO Box 416 Lundsiden, N-4604 Kristiansand,Norway.

^5^Vikersund Rehabilitation Center, Haaviks vei 25, N-3370 Vikersund, Norway.

^6^Hospital for Rheumatic Diseases Haugesund, PO Box 2175, N-5504 Haugesund, Norway.

^7^Rehabilitering Vest Rehabilitation Center, PO Box 2175, N-5504 Haugesund, Norway.

^8^Muritunet Rehabilitation Center, Grandedata 58, N-6210 Valldal, Norway.

^9^Valnesfjord Health Sports Center, Østerkløftveien 249, N-8215 Valnesfjord, Norway.

^10^Meråker Rehabilitation Center, Østigardsveien 24, N-7530 Meråker, Norway.

*Corresponding author. Correspondence: [anne-lene.svartrud@diakonsyk.no](mailto:anne-lene.svartrud@diakonsyk.no)

**Additional file 3.** Predefined hypotheses with rationale and results.

| **PART ONE Changes in median TOTAL PASS RATES (PRs)** | | | | | | | |
| --- | --- | --- | --- | --- | --- | --- | --- |
|  | | | Indicator | H_total_  number | **Hypotheses (H)** | **Proportion / PR**  **/change (**Δ**)** | **Confirmed (Yes/No)** |
| Center-reported QIs (structure) | | | C 01-  C 19 | 1 | For center-reported QIs, a moderate to high change score is expected for median total PR between T1 and T2. | medianPR_totalT1_=53%  medianPR_totalT2_=90%  Δ_T2-T1_=37%- points | **yes** |
| Patient-reported QI  (process and outcomes) | | | P 01-  P 14 | 2 | For patient-reported QIs, a small to moderate change score is expected for median total PR between T1 and T2. | medianPR_totalT1_=75%  medianPR_totalT2_=79%  Δ_T2-T1_=4%- points | **yes** |
| Patient-reported QIs (process in subgroups) | | | P 01-  P 11 | 3 | For process QIs, the change score for median total PR is expected to be small to moderate for both group 1 (inflammatory rheumatic disease) and group 2 (fibromyalgia) between T1 and T2.  (n_group1_=74-114) (n_group2_=14-40) | medianPR_processT1group1_=73%  medianPR_processT2group1_=82%  Δ_T2-T1group1_=9%- points  medianPR_processT1group2_=70%  medianPR_processT2group2_=73%  Δ_T2-T1group2_=3%- points | **yes** |
| Patient-reported QIs  (outcomes in subgroups) | | | P 12-  P 14 | 4 | For outcome QIs, the change score for the median total PR is expected to be zero or small for both group 1 (inflammatory rheumatic disease) and group 2 (fibromyalgia) between T1 and T2.  (n_group1_=74-114) (n_group2_=14-40) | medianPR_outcomeT1group1_=100%  medianPR_outcomeT2group1_=100%  Δ_T2-T1group1_=no change  medianPR_outcomeT1group2_=50%  medianPR_outcomeT2group2_=67%  Δ_T2-T1group2_=17%- points | no |
| Group 1= participants registered in the BRIDGE-trial with inflammatory rheumatic disease at admission.  Group 2= participants registered in the BRIDGE-trial with fibromyalgia or chronic widespread pain at admission. | | | | | | | |
| R  A  T  I  O  N  A  L  E | | Assumptions about the T1-situation  Seven out of 8 centers in the BRIDGE trial participated in the pilot study (1) concerning quality indicators in rehabilitation. The T1-situation may be similar to the situation in the pilot study as regards quality indicators (QIs) in rehabilitation. The results from the pilot study (1) showed:   - C01-C19_For structure items measured in specialist care, the median sum PR was 63%, ranging from 21% to 100% (answered by providers in the beginning of the test period). One among 15 units in specialist care reached a 100% sum PR. (This unit was not among participating units in the BRIDGE-trial) - P01-P14_For process and outcome items measured in specialist care, the mean sum PR was 72% (answered by patients one to two months after completion of a rehabilitation program at 14 units) (there were no patients from the last unit). Among the centers in the pilot, the value of the mean sum PR ranged from 36 to 89%. Looking separately on process and outcome items, the mean sum PR for process items (P01-P11) was 70% in specialist care. Box plots from the pilot study illustrate that the data concerning process QIs was not tightly grouped, but spread along a wide spectrum from zero to 100. For outcome items (P12-P14) the mean sum PR was 83% in specialist care. Data concerning outcome QIs was spread from zero to top, as well, but skewed towards the interval 67-100%. - In the pilot study, less optimal quality was found for both structure and process domains. Potential for improvement was found for important quality domains such as goal-directed evaluation along the rehabilitation process, individual planning of follow-up, involving external personnel, and use of validated assessment instruments at admission, discharge and 3-6 months after rehabilitation. - The largest diagnose groups in the BRIDGE-trial was inflammatory rheumatic disease (group 1) and fibromyalgia syndrome/chronic widespread pain (group2). Previous studies have shown that immediately after, or few weeks after, completion of similar multidisciplinary rehabilitation studies, improvements were found for both groups regarding health related quality of life, goal attainment and function (2, 3). In the pilot study, PRs for the outcome items were considerable high for both group 1 and group 2 (1)   *The table continues…* | | | | | |
| R  A  T  I  O  N  A  L  E | Expected influence of the BRIDGE-intervention on the total pass rates for quality in rehabilitation:   - In our study, providers of the BRIDGE-intervention are expected to strive for improved continuity in the whole rehabilitation process. Thus, the structural domain of quality in rehabilitation is expected to be influenced in form of providers who emphasize patient participation and information to externals, regarding individual goal setting, goal-directed rehabilitation plan and follow-up plan, evaluation using validated outcome measures and feedback. However, in order to tick “yes” for a structural item, two requirements have to be met: i) *included in the written procedures of the rehabilitation center*, and ii) *in daily use*. For some units, the development of new written procedures may appear as large changes and difficult to fulfill. When both requirements are not fulfilled, a unit is not allowed to change answer from “no” to “yes”. - Further, the process domain is expected to be influenced as well, in form of improved clinical practice at each unit reflected in the patients’ experiences. Patients report their experiences in the patient-reported QI-form. Compared to T1, we expect a small to moderate higher proportion of patients at T2 answering “yes” regarding experienced influence on their own goal setting, rehabilitation plan, follow-up plan, evaluation and feedback.   Summed up:  Elements in the BRIDGE-intervention match important quality indicators which were identified in the pilot study as with potential for improvement. Changes should be reflected in both system and user perspective of quality in rehabilitation. We therefore expect small to moderate changes between T1 and T2 in total mean pass rates for both questionnaires in the quality indicator set. For the subgroups (group 1 and group 2, respectively), we expect changes in process and outcomes to be comparable. | | | | | | |

| **PART TWO Single indicator pass rate (PR) change**   1. **PATIENT-PARTICIPATION IN GOAL SETTING AND REHABILITATION PROCESS** | | | | | | | | | | | | |
| --- | --- | --- | --- | --- | --- | --- | --- | --- | --- | --- | --- | --- |
| **QI no.** | | | **H no.** | | | **Hypotheses (H)**  (H_single_) | | **Proportion / N**  **/change (**Δ**)** | | | **Confirmed (Yes/No)** | |
| C01 | | | The user/patient shall participate in setting rehabilitation goals. | | | | | | | | | |
| P04 | | | Were you actively involved in setting specific goals for the rehabilitation period? | | | | | | | | | |
|  | | | 1 | | | C01: All participating centers who answered “yes” at T1 are expected to answer “yes” at T2. | | N_T1=yes_=6  N_yes_sustained_T1toT2_=6  Proportion_sustained_=100% | | | **yes** | |
|  | | | 2 | | | C01: The proportion of participating centers having a shift from “no” at T1 to “yes” at T2 is expected to be small to moderate. | | N_T1=no_=2  N_improved_=2  Δ_absolute_=25% (2/8) | | | **yes** | |
|  | | | 3 | | | P04: The proportion of patients answering “yes” at T2 is expected to be similar or slightly higher than proportion of patients answering “yes” at T1.  *(Proportion=N_participants who checked “yes”_/N_participants who checked “yes” or “no”)_* | | Proportion_T1_=159/162  Proportion_T2_=131/132  Δ_T2-T1_=99.2%-98.2%=1% | | | **yes** | |
| R  A  T  I  O  N  A  L  E | | | Assumptions about the T1-situation   - C01_Results from the pilot study (1), PRs (providers in specialist health care) = 93% - P04_Results from the pilot study (1), PRs (patients in specialist heath care) = 97% - C01_Six out of eight participating centers in the BRIDGE-study participated in previous studies involving goal setting in rehabilitation. Previous studies were the PRAISE study (center 7), The BRIDGE pilot study (centers 2, 5, 6 and 8), and both the PRAISE and the BRIDGE pilot study (center 4). Consequently, we have yet another reason for expecting patient involvement in goal setting as clinical practice already in the T1-situation. However, the request for patient involvement in goal setting as established in written procedures, may be more difficult to fulfill. We expect PRs higher than 6% when it comes to patient involvement in goal setting from the provider perspective at T1. - C01_Prior to the PRAISE study, 6/6 rehabilitation sites reported individual goals to be defined as part of regular practice, and at 5/6 of those sites, the goals were developed in collaboration between the patient and team member(s) (reported by the providers) (4, p 149) - C01_5/5 rehabilitation sites in Norway reported individual goals to be defined / developed together with team member(s) (5). As such, we have reasons to believe that this reflected also the regular clinical practice, but not necessarily the written procedures.   *The table continues…* | | | | | | | | | |
| R  A  T  I  O  N  A  L  E | | | Expected influence of the BRIDGE-intervention on this specific area of quality in rehabilitation:  Patient participation in goal setting at admission and discharge is included in the BRIDGE-intervention. At baseline, patient-specific rehabilitation goals will be developed by the patient together with the multidisciplinary team or the local coordinator. At discharge, patient-specific rehabilitation goals are developed in the same way, but now the contents in goals are facing the follow-up period at home (6, 7 (checklist item no. 3, 4, 6, 10)). Development of written procedure at each center does not directly follow from written guiding booklets for use in the restricted BRIDGE time period. Therefore, we do not expect high influence on the structural domain.  Summed up: The clinical practice of providers is likely to be affected by the intervention (T2) in the form of goal setting dialogues with every patient, but we do not know whether written procedures (if lacking) are developed (structural factors) as a consequence of the BRIDGE-intervention. Further, we do not know if all patients want to be involved in goal setting in the T2-situation, even if invited to participate. Because of expected high PRs already at T1, we consider C01 and P04 less likely to be further affected by the BRIDGE-intervention. | | | | | | | | | |
| C02 | | | The user/patient shall participate in planning his /her own rehabilitation process. | | | | | | | | | |
| C03 | | | A template is used to prepare an individual rehabilitation plan for the user/patient. | | | | | | | | | |
| P03 | | | Was a written plan developed for the rehabilitation period (comprising your rehabilitation goals, what you should practice etc.)? | | | | | | | | | |
| P05 | | | Where you actively involved in preparing a specific written plan for the rehabilitation period (mentioned in question 3)? | | | | | | | | | |
|  | | | **H no.** | | | **Hypotheses (H)**  (H_single_) | | **Proportion / N**  **/change (**Δ**)** | | | **Confirmed (Yes/No)** | |
|  | | | 4 | | | C02: All participating centers who answered “yes” at T1 are expected to answer “yes” at T2 | | N_T1=yes_=6  N_yes_sustained_T1toT2_=6  Proportion_sustained_=100% | | | **yes** | |
|  |  |  | 5 | | | C03: All participating centers who answered “yes” at T1 are expected to answer “yes” at T2 | | N_T1=yes_=5  N_yes_sustained_T1toT2_=5  Proportion_sustained_=100% | | | **yes** | |
|  | | | 6 | | | C02: The proportion of participating centers having a shift from “no” at T1 to “yes” at T2 is expected to be small to moderate. | | N_T1=no_=2  N_improved_=2  Δ_absolute_=25% (2/8) | | | **yes** | |
|  |  |  | 7 | | | C03: The proportion of participating centers having a shift from “no” at T1 to “yes” at T2 is expected to be small to moderate. | | N_T1=no_=3  N_improved_=3  Δ_absolute_=37.5% (3/8) | | | no | |
|  | | | 8 | | | P03: Proportion of patients answering “yes” at T2 is expected to be moderate higher than proportion of patients answering “yes” at T1. | | Proportion_T1_=148/161  Proportion_T2_=125/132  Δ_T2-T1_=94.7% -91.9%=2.8% | | | no | |
|  |  |  | 9 | | | P05: Proportion of patients answering “yes” at T2 is expected to be moderate higher than proportion of patients answering “yes” at T1. | | Proportion_T1_=140/160  Proportion_T2_=123/132  Δ_T2-T1_=93.2%-87.5%=5.7% | | | no | |
| *(Proportion=N_participants who checked “yes”_/N_participants who checked “yes” or “no”)_*  *The table continues…* | | | | | | | | | | | | |
| R  A  T  I  O  N  A  L  E | | | Assumptions about the T1-situation   - C02_Results from the pilot study (1), PRs (providers in specialist health care) = 93% - C03_Results from the pilot study (1), PRs (providers in specialist health care) = 79% - P03_Results from the pilot study (1), PRs (patients in specialist heath care) = 82% - P05_Results from the pilot study (1), PRs (patients in specialist heath care) = 76% - C02 _Prior to the PRAISE study, 2/6 rehabilitation sites reported patient involvement in development, adjustment and evaluation of rehabilitation plans (4, p 149). - C03 _ Prior to the PRAISE study, 3/6 rehabilitation sites reported use of individual rehabilitation plans with goals, action plan and cooperative agreements (4, p 149). - C03_5/5 rehabilitation sites in Norway reported that they included an individual rehabilitation plan. Data concerning patient involvement in preparing this plan is not included in the paper (5). This reflects the clinical practice, but not necessarily written procedures.     Expected influence of the BRIDGE-intervention on this specific area of quality in rehabilitation:  In the BRIDGE-intervention, the patient and the team member(s) develop a plan for actions and efforts for reaching patient-specific goals and improve the function. The planning comprises plans for self-management, as well as support from health care providers, next of kin or important others. A written individual rehabilitation plan is included in the BRIDGE-intervention. The template is available in the patients’ guiding booklets. The plan is developed by the patient and the team (or a professional who represents the team), and comprises the goals, appurtenant goal-directed interventions, responsible person/instance, and strategies for potential obstacles. (6, 7 (checklist item no. 7, 11, 12, 13)). We do not know if written material for use in the research period, results in written procedure at the actual centers.  Summed up:  Patient involvement in planning the rehabilitation period as well as clinical use of a template of a rehabilitation plan, are likely to be affected by the BRIDGE-intervention (T2). However, the influence may be better reflected in process indicators than structural indicators. | | | | | | | | | |
| C05 | | | There are at least two meetings between the user/patient and the interdisciplinary team, or between the user/patient and a professional who represents the interdisciplinary team*.* | | | | | | | | | |
| P06 | | | Did you participate in at least two meetings with the interdisciplinary team or a professional representing the team during which your goal(s) and goal attainment so far were discussed? | | | | | | | | | |
|  | | | H no | **Hypotheses (H)**  (H_single_) | | | | **Proportion / N**  **/change (**Δ**)** | | | **Confirmed (Yes/No)** | |
|  | | | 10 | C05: All participating centers who answered “yes” at T1 are expected to answer “yes” at T2 | | | | N_T1=yes_=6  N_yes_sustained_T1toT2_=6  Proportion_sustained_=100% | | | **yes** | |
|  | | | 11 | C05: The proportion of participating centers having a shift from “no” at T1 to “yes” at T2 is expected to be small to moderate. | | | | N_T1=no_=2  N_improved_=2  Δ_absolute_=25% (2/8) | | | **yes** | |
|  | | | 12 | P06: Proportion of patients answering “yes” at T2 is expected to be moderate higher than proportion of patients answering “yes” at T1.  *(Proportion=N_participants who checked “yes”_/N_participants who checked “yes” or “no”)_* | | | | Proportion_T1_=138/160  Proportion_T2_=127/131  Δ_T2-T1_=97%-86.3%=10.7% | | | no | |
| R  A  T  I  O  N  A  L  E | | | Assumptions about the T1-situation   - C05_Results from the pilot study (1), PRs (providers in specialist health care) = 71% - P06_Results from the pilot study (1), PRs (patients in specialist heath care) = 77% - C05 _Prior to the PRAISE study, 2/6 rehabilitation sites reported some patient participation in team meetings (4, p 149). - C05 _5/5 rehabilitation sites in Norway included patient participation in team meeting at admission/discharge (5). This reflects the clinical practice, but not necessarily written procedures.   Expected influence of the BRIDGE-intervention on this specific area of quality in rehabilitation:  In the BRIDGE-intervention, a goal-setting conversation between the patient and the multidisciplinary team (or a professional who represents the team) is mandatory. A telephone-based consultation in the follow-up period is mandatory, as well, addressing progress towards goals, adherence to self-management and, if applicable, necessary contacts and support from primary health (6, 7(checklist item no. 4, 18)). *The table continues…*  Additionally, meetings between the patient and the team (or a professional who represents the team) are expected in order to develop written rehabilitation plans, self-management plans and a plan for follow-up (6, 7 (checklist item no. 7, 11, 12, 13)).  Summed up:  Meetings between the patient and team member(s) are likely to be affected by the BRIDGE-intervention (T2). We expect this to be reflected in positive change score for both provider-reported and patient-reported QIs. However, the influence on provider-reported change score may be less due to the requirement concerning development of written procedure (beyond the written guiding booklets meant for the research period). Further, the influence on patient-reported change score may be less due to complex formulation of that question (how do the respondent understand the question?). | | | | | | | | | |
| C04 | | | The user/patient shall participate in evaluating his/her ongoing process. | | | | | | | | | |
|  | | | H no. | | | **Hypotheses (H)**  (H_single_) | | **Proportion / N**  **/change (**Δ**)** | | **Confirmed (Yes/No)** | | |
|  | | | 13 | | | C04: All participating centers who answered “yes” at T1 are expected to answer “yes” at T2 | | N_T1=yes_=5  N_yes_sustained_T1toT2_=5  Proportion_sustained_=100% | | **yes** | | |
|  | | | 14 | | | C04: The proportion of participating centers having a shift from “no” at T1 to “yes” at T2 is expected to be small to moderate. | | N_T1=no_=3  N_improved_=3  Δ_absolute_=37.5% (3/8) | | no | | |
| R  A  T  I  O  N  A  L  E | | | Assumptions about the T1-situation   - C04_Results from the pilot study (1), PRs (providers in specialist health care) = 93% - C04 _Prior to the PRAISE study, 2/6 rehabilitation sites reported patient involvement in evaluation during the stay (4, p 149).   Expected influence of the BRIDGE-intervention on this specific area of quality in rehabilitation:  According to the protocol, the BRIDGE trial is expected to empower patients to monitor and evaluate their health, function and their rehabilitation progress / goal progress over time (6). In the BRIDGE-intervention, the patient and the multidisciplinary team evaluate the ongoing goal process, and they make adjustments (if needed) during the rehabilitation stay. The patient and a member of the team make a new evaluation of the goal process during the mandatory telephone-based consultation in the follow-up period. Agreed adjustments of plans, efforts or actions are implemented (if needed) (6, 7 (checklist item no.9, 14, 18-21)). Additionally, patients can monitor their own progress when they log in to an electronic portal and complete the rehabilitation core set of outcomes five times over a one-year period. An auto-generated digital report visualizes the participant’s current status in health and function, and changes over time at each of the five reporting times (7 (checklist items 15-17).  Summed up:  Patient participation in ongoing evaluation of the goal-directed rehabilitation process is likely to be affected by the BRIDGE-intervention (T2). However, the influence on the structural domain may be less due to the requirement concerning development of written procedure (beyond the written guiding booklets). Consequently, we expect low to moderate (instead of moderate to high) influence. | | | | | | | | | |
| 1. **FOLLOW-UP PLAN AND CONTINUITY ACROSS LEVELS OF CARE** | | | | | | | | | | | | |
| **QI no.** |  | | | | | | | | | | | |
| C09 | The user/patient shall participate in preparing a specified written follow-up plan (aside from the epicrisis) for the follow-up process after the rehabilitation period. This plan shall also include the user's / patient's own efforts to maintain or improve function/health. | | | | | | | | | | | |
| C10 | If there is a need for healthcare support after the rehabilitation period, the relevant personnel are to be informed about the plan or participate in the development of the follow-up plan. | | | | | | | | | | | |
| P09 | Apart from regular epicrisis, was a written plan developed for the period after rehabilitation, including what you were expected to work on yourself?  (if you have answered “yes” to question number 7, go to question number 8. If you have answered “no” to question number 7, go to question number 9) | | | | | | | | | | | |
| P10 | Did you participate in developing the plan (question number 7)? *(AL: the written follow-up plan)* | | | | | | | | | | | |
| P11 | As a part of this plan, were you consulted as to whether you needed follow-up from healthcare or vocational professionals (NAV) or other personnel after the rehabilitation period?  *The table continues…* | | | | | | | | | | | |
|  | **H**  **no.** | | | | **Hypotheses (H)**  (H_single_) | | **Proportion / N**  **/change (**Δ**)** | | | | | **Confirmed (Yes/No)** |
|  | 15 | | | | C09: All participating centers who answered “yes” at T1 are expected to answer “yes” at T2 | | N_T1=yes_=5  N_yes_sustained_T1toT2_=5  Proportion_sustained_=100% | | | | | **yes** |
|  | 16 | | | | C09: The proportion of participating centers having a shift from “no” at T1 to “yes” at T2 is expected to be small to moderate. | | N_T1=no_=3  N_improved_=3  Δ_absolute_=37.5% (3/8) | | | | | no |
|  | 17 | | | | C10: All participating centers who answered “yes” at T1 are expected to answer “yes” at T2 | | N_T1=yes_=3  N_yes_sustained_T1toT2_=3  Proportion_sustained_=100% | | | | | **yes** |
|  | 18 | | | | C10: The proportion of participating centers having a shift from “no” at T1 to “yes” at T2 is expected to be moderate to high. | | N_T1=no_=5  N_improved_=5  Δ_absolute_=62.5% (5/8) | | | | | **yes** |
|  | 19 | | | | P09: Proportion of patients answering “yes” at T2 is expected to be slightly to moderate higher than proportion of patients answering “yes” at T1. | | Proportion_T1_=110/159  Proportion_T2_=107/132  Δ_T2-T1_=81.1%-69.2% =11.9% | | | | | **yes** |
|  | 20 | | | | P10: Proportion of patients answering “yes” at T2 is expected to be slightly to moderate higher than proportion of patients answering “yes” at T1. | | Proportion_T1_=106/110  Proportion_T2_=105/107  Δ_T2-T1_=98.1%-96.4% =1.7% | | | | | **yes** |
|  | 21 | | | | P11: Proportion of patients answering “yes” at T2 is expected to be slightly to moderate higher than proportion of patients answering “yes” at T1. | | Proportion_T1_=55/110  Proportion_T2_=74/106  Δ_T2-T1_=69.8%-50% =19.8% | | | | | **yes** |
| *(Proportion=N_participants who checked “yes”_/N_participants who checked “yes” or “no”)_* | | | | | | | | | | | | |
| R  A  T  I  O  N  A  L  E | Assumptions about the T1-situation   - C09_Results from the pilot study (1), PRs (providers in specialist health care) = 36% - C10_Results from the pilot study (1), PRs (providers in specialist health care) = 36% - P09_Results from the pilot study (1), PRs (patients in specialist heath care)= 71% - P10_Results from the pilot study (1), PRs (patients in specialist heath care)= 83% - P11_Results from the pilot study (1), PRs (patients in specialist heath care)= 23% - C09 and C10 _Prior to the PRAISE study, 0/6 rehabilitation sites reported use of a standard for follow-up management (4, p 149). - C09 and C10 _0/5 rehabilitation sites in Norway reported use of a standard for follow-up management (5).   Expected influence of the BRIDGE-intervention on this specific area of quality in rehabilitation:  In the BRIDGE-intervention, the patient and team member(s) plan relevant follow-up care according to patient’s need and available resources in the municipality (6). An individual specified written follow-up plan is included in the BRIDGE-intervention (6, 7 (checklist item no. 13)), as well as a plan for the patient’s self-management (6, 7 (checklist item no.12)). The follow-up plan is tailored according to patient’s needs and available resources in the municipality (6). Patient participation in development of a follow-up plan and plan for self-management is included in the BRIDGE-intervention (6, 7 (checklist item 12-13)).  Potential follow-up care is proposed in the protocol, as well as in electronic portal (multiple choice list for ticking off the most relevant services for each individual), and in the guiding booklets for health personnel and patients. Options to consider are: health services from primary care (like general practitioner, physiotherapy/occupational therapist), the patient’s employer / employees’ health services, the Norwegian Labour and Welfare Administration (NAV/concerning employment and benefits), Healthy Life Centers and local branches of various Patient Associations. (6, 7 (checklist item no. 13), 8). Special note about C10: There is no item in the checklist concerning information about the follow-up plan to relevant personnel subsequent the rehabilitation stay / participation for relevant follow-care in development of the follow-up plan.  *The table continues…*  However, the BRIDGE-intervention aims to enhance communication and continuity in rehabilitation across levels of care.  In consequence, we expect that considerations of dialogue and collaboration with follow-up care are implicit in checklist item no.11 (rehabilitation plan for the first period in the home setting), no.13 (planned follow-up care), no.20 (evaluate necessary follow-up in local environment), and no. 21 (ensuring necessary contacts and support in the patient’s home setting is established) (7).  Need of follow-up care are expected to vary in the patient group. Some patients with complex conditions and/or life situations are associated with follow-up plans developed in close collaboration with primary health care. Cases with minor complexity are associated with a plain, informative referral to follow-up care (further dialogue is not needed). Some patients contact relevant follow-up care by themselves, others need support from personnel from secondary care to ensure necessary contact and support from primary care. For others, the mandatory telephone call is sufficient follow-up for maintaining goal-directed rehabilitation plan and self-management (6).  Summed up:  Patient participation in developing a written follow-up plan and plan for self-management is likely to be affected by the BRIDGE-intervention (T2). We also expect shared decision-making in the evaluation of needs from necessary services after the rehabilitation period (T2), and information to relevant personnel about the follow up plan. However, in some cases the health professionals may evaluate the need for follow-up without including the patient perspective. In other cases, some patients do not need external services and only include support from the rehabilitation unit in the follow-up plan. Additionally, the influence on the structural domain may be less due to the request concerning development of written procedure (beyond the written guiding booklets). | | | | | | | | | | | |
| C06 | The user/patient is asked before meetings if he/she wants their next of kin to attend any of the meetings. | | | | | | | | | | | |
| P07 | Were you asked if you wanted your next of kin to attend any of the meetings? | | | | | | | | | | | |
|  | **H**  **no.** | | | | **Hypotheses (H)**  (H_single_) | | **Proportion / N**  **/change (**Δ**)** | | | | | **Confirmed (Yes/No)** |
|  | 22 | | | | C06: All participating centers who answered “yes” at T1 are expected to answer “yes” at T2 | | N_T1=yes_=1  N_yes_sustained_T1toT2_=1  Proportion_sustained_=100% | | | | | **yes** |
|  | 23 | | | | C06: The proportion of participating centers having a shift from “no” at T1 to “yes” at T2 is expected to be zero or small. | | N_T1=no_=7  N_improved_=0  Δ_absolute_=0 (0/8) | | | | | **yes** |
|  | 24 | | | | P07: Proportion of patients answering “yes” at T2 is expected to be equal or slightly higher than proportion of patients answering “yes” at T1.  *(Proportion=N_participants who checked “yes”_/N_participants who checked “yes” or “no”)_* | | Proportion_T1_=16/160  Proportion_T2_=20/131  Δ_T2-T1_=15.3%-10% =5.3% | | | | | **yes** |
| R  A  T  I  O  N  A  L  E | Assumptions about the T1-situation   - C06_Results from the pilot study (1), PRs (providers in specialist health care) = 57% - P07_Results from the pilot study (1), PRs (patients in specialist heath care) = 16% - C06 _Prior to the PRAISE study, 1/6 rehabilitation sites reported standard for family involvement (4, p 149). - C06 _0/5 rehabilitation sites in Norway reported standard for family involvement, but all the sites (5/5) reported family involvement based on indication (5).   Expected influence of the BRIDGE-intervention on this specific area of quality in rehabilitation:  No items in the checklist comprise invitation to meetings for family/friends (7). On the other hand, efforts and actions to ensure necessary contacts and support in the patient’s home setting are emphasized in the BRIDGE-trial protocol (6). In the protocol and in the guiding booklet for clinicians, family and friends are described as *important others* and *relevant support* to facilitate self-management-strategies, lifestyle changes and goal directed rehabilitation process (6, 8). Further, “planned support from others” is included as an item in the template for the written rehabilitation plan (6, 8).  Summed up:  Involvement of next of kin in meetings is less likely to be affected by the BRIDGE-program. We expect similar or slightly higher PRs for indicator C06 and P07, respectively, due to the BRIDGE-intervention. The influence of BRIDGE-intervention may be moderated down due to dominance from health professionals who may make decisions about patients’ need of involvement from next of kin, without asking the patients about it (reflected in expected low pass rate for P07). Additionally, the influence on the structural domain (C06) may also be moderated down due to the request concerning development of written procedure (beyond the written guiding booklets).  *The table continues…* | | | | | | | | | | | |
| C07 | The user/patient is asked before meetings if he/she wants some of the professionals he/she will relate to after the rehabilitation to attend any of the meetings. This may include a physiotherapist, general practitioner or a person from work if participating in vocational rehabilitation. | | | | | | | | | | | |
| P08 | Were you asked if you wanted professionals you will relate to after the rehabilitation period to attend any of the meetings, such as physiotherapist, your general practitioner, the labour and welfare administration (NAV) or a person from work, if you are participating in vocational rehabilitation? | | | | | | | | | | | |
|  | **H**  **no.** | | | | **Hypotheses (H)**  (H_single_) | | | | **Proportion / N**  **/change (**Δ**)** | | | **Confirmed (Yes/No)** |
|  | 25 | | | | C07: All participating centers who answered “yes” at T1 are expected to answer “yes” at T2 | | | | N_T1=yes_=0  N_yes_sustained_T1toT2_=0  Proportion_sustained_=0 | | | *Not applicable hypotheses* |
|  | 26 | | | | C07: The proportion of participating centers having a shift from “no” at T1 to “yes” at T2 is expected to be zero to small. | | | | N_T1=no_=8  N_improved_=1  Δ_absolute_=12.5% (1/8) | | | **yes** |
|  | 27 | | | | P08: Proportion of patients answering “yes” at T2 is expected to be equal or slightly higher than proportion of patients answering “yes” at T1.  *(Proportion=N_participants who checked “yes”_/N_participants who checked “yes” or “no”)_* | | | | Proportion_T1_=8/160  Proportion_T2_=17/131  Δ_T2-T1_=13%-5% =8% | | | **yes** |
| R  A  T  I  O  N  A  L  E | Assumptions about the T1-situation   - C07_Results from the pilot study (1), PRs (providers in specialist health care) = 57% - P08_Results from the pilot study (1), PRs (patients in specialist heath care) = 16% - C07 _Prior to the PRAISE study, 0/6 rehabilitation sites reported standard for follow-up management (4, p 149). - C07 _0/5 rehabilitation sites in Norway reported standard for follow-up management (5).   Expected influence of the BRIDGE-intervention on this specific area of quality in rehabilitation:  No items in the checklist comprise invitation to meetings for important others from primary health/other relevant services or instances. On the other hand, efforts and actions to ensure necessary contacts and support in the patient’s home setting are emphasized in the BRIDGE-trial protocol. Several instances and resources in the municipality are mentioned in the protocol as relevant support, for instance general practitioner, physiotherapy/occupational therapy in primary health care, the patient’s employer / employees’ health services, the Norwegian Labour and Welfare Administration (NAV/concerning employment and benefits), Healthy Life Centers and local branches of various Patient Associations (6). Contact with important professionals for the rehabilitation process after discharge is expected, but probably through phone calls or written referrals, and only extraordinarily through meetings.  Summed up: *As for involvement of next of kin:* Involvement of external professionals/important others in meetings is less likely to be affected by the BRIDGE-program. We expect similar or slightly higher PRs for indicator C07 and P08, respectively, due to the BRIDGE-intervention. The influence of BRIDGE-intervention may be moderated down due to dominance from health professionals who may make decisions about patients’ need of involvement from external professionals/important others, without asking the patients about it (reflected in expected low PR for P08). Additionally, the influence on the structural domain (C07) may also be moderated down due to the request concerning development of written procedure (beyond the written guiding booklets), and also due to other contact forms than meetings. | | | | | | | | | | | |
| 1. **ASSESSMENT, OUTCOMES AND TIME-POINTS OF EVALUATION** | | | | | | | | | | | | |
| **QI no.** | |  | | | | | | | | | | |
| P01 | | Where your health condition and life situation assessed during the first days of your rehabilitation period? (Answer “no” if both aspects were not assessed)  (If you have answered yes to question number 1, go to question number 2. If you have answered no to question number 1, go to question number 3). | | | | | | | | | | |
| P02 | | Did the assessments include both a physical examination and questions about mental and social conditions, network, home situation and – if relevant – your work situation?  *The table continues…* | | | | | | | | | | |
|  | | **H**  **no.** | | | **Hypotheses (H)**  (H_single_) | | **Proportion / N**  **/change (**Δ**)** | | | | | **Confirmed (Yes/No)** |
|  | | 28 | | | P01: Proportion of patients answering “yes” at T2 is expected to be similar or slightly higher than the proportion of patients answering “yes” at T1.  *(Proportion=N_participants who checked “yes”_/N_participants who checked “yes” or “no”)_* | | Proportion_T1_=153/162  Proportion_T2_=125/132  Δ_T2-T1_=94.7%-94.4% =0.3% | | | | | **yes** |
|  | | 29 | | | P02: Proportion of patients answering “yes” at T2 is expected to be similar or slightly higher than the proportion of patients answering “yes” at T1.  *(Proportion=N_participants who checked “yes”_/N_participants who checked “yes” or “no”)_* | | Proportion_T1_=136/153  Proportion_T2_=115/124  Δ_T2-T1_=92.7%-88.9% =3.8% | | | | | **yes** |
| R  A  T  I  O  N  A  L  E | | Assumptions about the T1-situation   - P01_Results from the pilot study (1), PRs (patients in specialist heath care) = 98% - P02_Results from the pilot study (1), PRs (patients in specialist heath care) = 87% - All participating centers in the BRIDGE-trial provide the rehabilitation service through multidisciplinary teams. - Prior to the PRAISE study, 6/6 rehabilitation sites reported rehabilitation teams involving 4 professionals or more, and 6/6 rehabilitation sites reported standardized assessment on admission. (4, p 149). - 5/5 rehabilitation sites in Norway reported standardized assessment at admission in the paper of Grotle (5). This standardized assessment includes comprehensive, bio-psycho-social assessment and evaluation at rehabilitation sites for people with rheumatic and musculosekleta disesases (RMDs) in Norway (5, 9). Typical assessment includes various outcomes, such as health status, symptoms, body function/structure, activity and participation, personal factors and health related quality of life (5, 9)   Expected influence of the BRIDGE-intervention on this specific area of quality in rehabilitation:  No items in the checklist comprise recommended content of the assessment of health condition and life situation during the first days of the rehabilitation stay. On the other hand,  the BRIDGE-trial is based on a biopsychosocial understanding of health and functioning, implying complex interactions between a person’s body structure and functions, activity performance, social participation, and the individual personality and environment (6). As a consequence, a holistic and systematic approach to the analysis of a patient’s disability is emphasized in the BRIDGE-trial protocol (6), and the relevance of physical, mental and social conditions are reflected in the rehabilitation core set outcome measures used in the BRIDGE-trial (a mandatory core set (10) , included in the electronic portal). Following domains are central in the BRIDGE-trial for providing information about current status, development over time, and for evaluating rehabilitation: physical function, pain, fatigue, mental health, social participation, daily activities, health-related quality of life, coping, motivation, and goal attainment. Rehabilitation interventions tailored to patient-specific goals and the individual’s unique home setting and life situation are emphasized in the BRIDGE-trial protocol (6)*.* Interventions tailored to patient’s needs and available resources in the community imply a comprehensive assessment approach (6). However, a bio-psycho-social assessment is expected also at T1.  Summed up: The BRIDGE-intervention facilitates an initial bio-psycho-social assessment, but the same approach to multidisciplinary, holistic assessment of health condition and life situation is expected also at T1. | | | | | | | | | | |
| C08 | | The rehabilitation unit uses reliable^***^ questionnaires and/or functional tests to assess physical, mental and/or social conditions.  (***Reliable questionnaires and/or functional tests = quality assured/validated questionnaires and/or functional tests) | | | | | | | | | | |
| C11 | | The user’s/patient’s goal / goal attainment is to be assessed with a reliable instrument. | | | | | | | | | | |
| C14 | | The user’s/patient’s function is to be registered using a reliable instrument. | | | | | | | | | | |
| C17 | | The user’s/patient’s health-related quality of life is to be assessed using a reliable instrument. | | | | | | | | | | |
|  | | **H**  **no.** | | | **Hypotheses (H)** | | | | **Proportion / N**  **/change (**Δ**)** | | | **Confirmed (Yes/No)** |
|  | | 30 | | | C08: All participating centers who answered “yes” at T1 are expected to answer “yes” at T2. | | | | N_T1=yes_=8  N_yes_sustained_T1toT2_=8  Proportion_sustained_=100% | | | **yes** |
|  |  | 31 | | | C11: All participating centers who answered “yes” at T1 are expected to answer “yes” at T2. | | | | N_T1=yes_=1  N_yes_sustained_T1toT2_=1  Proportion_sustained_=100% | | | **yes** |
|  |  | *The table continues…* | | | | | | | | | | |
|  |  | 32 | | | C14: All participating centers who answered “yes” at T1 are expected to answer “yes” at T2. | | | | N_T1=yes_=8  N_yes_sustained_T1toT2_=8  Proportion_sustained_=100% | | | **yes** |
|  |  | 33 | | | C17: All participating centers who answered “yes” at T1 are expected to answer “yes” at T2. | | | | N_T1=yes_=2  N_yes_sustained_T1toT2_=2  Proportion_sustained_=100% | | | **yes** |
|  |  | 34 | | | C08: All of the participating centers who answered “no” at T1 are expected to answer “yes” at T2. | | | | N_T1=no_=0  N_improved_=0  Proportion_improved_=0 | | | *Not applicable hypotheses* |
|  |  | 35 | | | C11: All of the participating centers who answered “no” at T1 are expected to answer “yes” at T2. | | | | N_T1=no_=7  N_improved_=7  Proportion_improved_=100% | | | **yes** |
|  |  | 36 | | | C14: All of the participating centers who answered “no” at T1 are expected to answer “yes” at T2. | | | | N_T1=no_=0  N_improved_=0  Proportion_improved_=0 | | | *Not applicable hypotheses* |
|  |  | 37 | | | C17: All of the participating centers who answered “no” at T1 are expected to answer “yes” at T2. | | | | N_T1=no_=6  N_improved_=6  Proportion_improved_=100% | | | **yes** |
|  |  | 38 | | | C08: The change-score for C08 is expected to be small to moderate. | | | | PR_T1_=100%  PR_T2_=100%  Δ_absolute_=0 | | | *Not applicable hypotheses* |
|  |  | 39 | | | C11: The change-score for C11 is expected to be moderate to high. | | | | PR_T1_=12.5%  PR_T2_=100%  Δ_absolute_=87.5% | | | **yes** |
|  |  | 40 | | | C14: The change-score for C14 is expected to be small. | | | | PR_T1_=100%  PR_T2_=100%  Δ_absolute_=0 | | | *Not applicable hypotheses* |
|  |  | 41 | | | C17: The change-score for C17 is expected to be moderate to high. | | | | PR_T1_=25%  PR_T2_=100%  Δ_absolute_=75% | | | **yes** |
| R  A  T  I  O  N  A  L  E | | Assumptions about the T1-situation   - C08_Results from the pilot study (1), PRs (patients in specialist heath care) = 86% - C11_Results from the pilot study (1), PRs (patients in specialist heath care) = 43% - C14_Results from the pilot study (1), PRs (patients in specialist heath care) = 93% - C17_Results from the pilot study (1), PRs (patients in specialist heath care) = 43% - Prior to the PRAISE study, 6/6 rehabilitation sites reported standardized assessment on admission (4, p 149). - 5/5 rehabilitation sites in Norway reported standardized assessment at admission in the paper of Grotle (5).   Expected influence of the BRIDGE-intervention on this specific area of quality in rehabilitation:  The digital assessment solution provides a controlled use of assessment tools in BRIDGE. The assessment plans are identical for all participating rehabilitation units.  Selected assessment tool in the BRIDGE-trail is a Norwegian core set of patient-reported outcome measures for use in multidisciplinary rehabilitation (10).  *The table continues…*  The core set covers aspects of physical, mental and social function and consists of one performance-based test for physical function (30-second Sit to Stand test) and the following patient reported outcome measures: Numeric rating scale of fatigue and pain, Patient-Specific Functional Scale (patient’s goal and goal attainment), Hannover Functional Questionnaire, Effective Musculoskeletal Consumer Scale, Hopkins Symptoms Checklist 5-item version, the social participation score from the COOP/WONCA, and the 5-level EuroQol 5 Dimensions (health related quality of life) (10). All instruments in the core set are reliable and validated in RMDs population (10) (6, 7 (checklist item no. 6,10,15,16))  Summed up:  Use of quality assured/validated questionnaires and/or functional tests are likely to be affected by the BRIDGE-intervention, due to a controlled use of assessment tools in the electronic portal. | | | | | | | | | | |
| C12 | | The user’s/patient’s goal / goal attainment is to be assessed with a reliable instrument at the beginning and the end of the rehabilitation period. | | | | | | | | | | |
| C15 | | The user’s/patient’s function is to be registered using a reliable instrument at the beginning and the end of the rehabilitation period. | | | | | | | | | | |
| C18 | | The user’s/patient’s health-related quality of life is to be assessed using a reliable instrument at the beginning and the end of the rehabilitation period. | | | | | | | | | | |
|  | | **H**  **no.** | | | **Hypotheses (H)**  (H_single_) | | | | **Proportion / N**  **/change (**Δ**)** | | | **Confirmed (Yes/No)** |
|  | | 42 | | | C12: All participating centers who answered “yes” at T1 are expected to answer “yes” at T2. | | | | N_T1=yes_=7  N_yes_sustained_T1toT2_=7  Proportion_sustained_=100% | | | **yes** |
|  | | 43 | | | C15: All participating centers who answered “yes” at T1 are expected to answer “yes” at T2. | | | | N_T1=yes_=8  N_yes_sustained_T1toT2_=8  Proportion_sustained_=100% | | | **yes** |
|  | | 44 | | | C18: All participating centers who answered “yes” at T1 are expected to answer “yes” at T2. | | | | N_T1=yes_=2  N_yes_sustained_T1toT2_=2  Proportion_sustained_=100% | | | **yes** |
|  | | 45 | | | C12: All of the participating centers who answered “no” at T1 are expected to answer “yes” at T2. | | | | N_T1=no_=1  N_improved_=1  Proportion_improved_=100% | | | **yes** |
|  | | 46 | | | C15: All of the participating centers who answered “no” at T1 are expected to answer “yes” at T2. | | | | N_T1=no_=0  N_improved_=0  Proportion_improved_=0 | | | *Not applicable hypotheses* |
|  | | 47 | | | C18: All of the participating centers who answered “no” at T1 are expected to answer “yes” at T2. | | | | N_T1=no_=6  N_improved_=6  Proportion_improved_=100% | | | **yes** |
|  | | 48 | | | C12: The change-score for C12 is expected to be moderate to high. | | | | PR_T1_=87.5%  PR_T2_=100%  Δ_absolute_=12.5% | | | no |
|  | | 49 | | | C15: The change-score for C15 is expected to be small. | | | | PR_T1_=100%  PR_T2_=100%  Δ_absolute_=0 | | | *Not applicable hypotheses* |
|  | | 50 | | | C18: The change-score for C18 is expected to be moderate to high. | | | | PR_T1_=25%  PR_T2_=100%  Δ_absolute_=75% | | | **yes** |
| R  A  T  I  O  -  N  A  L  E | | Assumptions about the T1-situation   - C12_Results from the pilot study (1), pass rates (patients in specialist heath care) = 43% - C15_Results from the pilot study (1), pass rates (patients in specialist heath care) = 93% - C18_Results from the pilot study (1), pass rates (patients in specialist heath care) = 36%   *The table continues…*  Expected influence of the BRIDGE-intervention on this specific area of quality in rehabilitation:  In the BRIDGE-trial, the instrument *Patient-Specific Functional Scale* *(PSFS)* is primary outcome, used to evaluate patient’s goals and goal attainment. 5-level EuroQol 5 Dimensions is secondary outcome, used to evaluate health related quality of life. 30-second Sit to Stand test is secondary outcome, used to evaluate physical function. Further, the following instruments complete the standardized assessment and evaluation in the BRIDGE-trial; Numeric rating scale of fatigue and pain, Hannover Functional Questionnaire, Effective Musculoskeletal Consumer Scale, Hopkins Symptoms Checklist 5-item version, and the social participation score from the COOP/WONCA. All instruments in the core set are reliable and validated in RMDs population (10). Patients complete the core set at admission and discharge of the rehabilitation stay, and after 2, 7 and 12 months (6, 7 (checklist item no. 6, 10, 16)).  Summed up:  Use of quality assured/validated questionnaires and/or functional tests at the beginning and end of the rehabilitation period, are likely to be affected by the BRIDGE-intervention, due to a controlled use of assessment tools in the electronic portal. | | | | | | | | | | |
| C13 | | The user’s/patient’s goal / goal attainment is to be assessed with a reliable instrument 3-6 months after the rehabilitation period. | | | | | | | | | | |
| C16 | | The user’s/patient’s function is to be registered with a reliable instrument 3-6 months after the rehabilitation period. | | | | | | | | | | |
| C19 | | The user’s/patient’s health-related quality of life is to be assessed using a reliable instrument 3-6 months after the rehabilitation period. | | | | | | | | | | |
|  | | **H**  **no.** | | | **Hypotheses (H)**  (H_single_) | | | | **Proportion / N**  **/change (**Δ**)** | | | **Confirmed (Yes/No)** |
|  | | 51 | | | C13: All participating centers who answered “yes” at T1 are expected to answer “yes” at T2. | | | | N_T1=yes_=1  N_yes_sustained_T1toT2_=1  Proportion_sustained_=100% | | | **yes** |
|  | | 52 | | | C16: All participating centers who answered “yes” at T1 are expected to answer “yes” at T2. | | | | N_T1=yes_=0  N_yes_sustained_T1toT2_=0  Proportion_sustained_=0 | | | *Not applicable hypotheses* |
|  | | 53 | | | C19: All participating centers who answered “yes” at T1 are expected to answer “yes” at T2. | | | | N_T1=yes_=0  N_yes_sustained_T1toT2_=0  Proportion_sustained_=0 | | | *Not applicable hypotheses* |
|  | | 54 | | | C13: All of the participating centers who answered “no” at T1 are expected to answer “yes” at T2. | | | | N_T1=no_=7  N_improved_=7  Proportion_improved_=100% | | | **yes** |
|  | | 55 | | | C16: All of the participating centers who answered “no” at T1 are expected to answer “yes” at T2. | | | | N_T1=no_=8  N_improved_=8  Proportion_improved_=100% | | | **yes** |
|  | | 56 | | | C19: All of the participating centers who answered “no” at T1 are expected to answer “yes” at T2. | | | | N_T1=no_=6  N_improved_=6  Proportion_improved_=100% | | | **yes** |
|  | | 57 | | | C13: The change-score for C13 is expected to be moderate to high. | | | | PR_T1_=12.5%  PR_T2_=100%  Δ_absolute_=87.5% | | | **yes** |
|  | | 58 | | | C16: The change-score for C16 is expected to be moderate to high. | | | | PR_T1_=0  PR_T2_=100%  Δ_absolute_=100% | | | **yes** |
|  | | 59 | | | C19: The change-score for C19 is expected to be moderate to high. | | | | PR_T1_=0  PR_T2_=100%  Δ_absolute_=100% | | | **yes** |
| R  A  T  I  O  N  A  L  E | | *The table continues…*  Assumptions about the T1-situation   - C13_Results from the pilot study (1), PRs (patients in specialist heath care) = 21% - C16_Results from the pilot study (1), PRs (patients in specialist heath care) = 29% - C19_Results from the pilot study (1), PRs (patients in specialist heath care) = 21%   Expected influence of the BRIDGE-intervention on this specific area of quality in rehabilitation:  No items in the checklist comprise recommendations concerning data collection for clinical assessments 3-6 months after the rehabilitation stay. On the other hand, item 16 in the check list underlines that providers of rehabilitation have to ensure that patients know how to log in to electronic portal for new assessments after 2, 7 and 12 months, from home (7). Further, support and follow-up more than 3 months are emphasized in the BRIDGE-protocol in order to obtain successful rehabilitation, explained by the nature of life-style changes and behavior changes (6). Health-related lifestyle changes often involve both cognitive and behavioral elements. The rehabilitation process may be characterized as unclear and fragile in the first months. Many patients need support over a longer time period to be able to settle new habits in everyday life (6). The point of longer time frames is also reflected in the mandatory follow-up consultation by phone about 4 weeks after discharge, with the possibility of 3 further consultations by phone during the first 6 months at home (6, 7 (item 14, 18-19)).  Summed up:  Use of quality assured/validated questionnaires and/or functional tests 3-6 months after the rehabilitation period, is likely to be affected by the BRIDGE-intervention, due to a controlled use of assessment tools in the electronic portal. | | | | | | | | | | |
| P12 | | As a result of the rehabilitation period, have you achieved one or several goals that are important to you? | | | | | | | | | | |
| P13 | | As a result of the rehabilitation period, have you achieved an improvement in your physical, mental and/or social functioning that is important to you? | | | | | | | | | | |
| P14 | | As a result of the rehabilitation, do you think your quality of life has improved? | | | | | | | | | | |
|  | | **H**  **no.** | | | **Hypotheses (H)**  (H_single_) | | | | **Proportion / N**  **/change (**Δ**)** | | | **Confirmed (Yes/No)** |
|  | | 60 | | | P12: Proportion of patients answering “yes” at T2 is expected to be equal to (or slightly higher than) proportion of patients answering “yes” at T1. | | | | Proportion_T1_=139/161  Proportion_T2_=110/132  Δ_T2-T1_=83.3%-85.7% = -2.4% (negative change) | | | no |
|  | | 61 | | | P13: Proportion of patients answering “yes” at T2 is expected to be equal to (or slightly higher than) proportion of patients answering “yes” at T1. | | | | Proportion_T1_=124/161  Proportion_T2_=102/132  Δ_T2-T1_=77.3%-77%=0.3% | | | **yes** |
|  | | 62 | | | P14: Proportion of patients answering “yes” at T2 is expected to be equal to (or slightly higher than) proportion of patients answering “yes” at T1. | | | | Proportion_T1_=129/161  Proportion_T2_=95/132  Δ_T2-T1_=72%-80.1% = -8.1% (negative change) | | | no |
| *(Proportion=N_participants who checked “yes”_/N_participants who checked “yes” or “no”)_* | | | | | | | | | | | | |
| R  A  T  I  O  N  A  L  E | | Assumptions about the T1-situation   - P12_Results from the pilot study (1), PRs (patients in specialist heath care)= 92% - P13_Results from the pilot study (1), PRs (patients in specialist heath care)= 89% - P14_Results from the pilot study (1), PRs (patients in specialist heath care)= 77% - At discharge in the PRAISE-study, a significant treatment effect was found on health related quality of life. After six or twelve months, the positive changes after rehabilitation gradually declined. However, the outcome values were maintained at higher levels in the follow up period compared to baseline. This was the situation for both the control-group and the intervention group in the PRAISE-study (3).   Expected influence of the BRIDGE-intervention on this specific area of quality in rehabilitation:  Elements in the BRIDGE-program may influence and improve goal attainment, physical function, health-related quality of life, emotional status and other psychosocial outcomes. The elements are likely to facilitate self-regulation strategies and promote health-related behavior changes (6). However, we expect this type of influence also at T1.  Summed up:  Two months after start of the rehabilitation stay, we expect patients’ outcomes to be likely affected by the BRIDGE-intervention. However, a similar influence is expected at T1 as well. | | | | | | | | | | |

**References**

1. Johansen I, Klokkerud M, Anke A, Børke J-B, Glott T, Hauglie U, et al. A quality indicator set for use in rehabilitation team care of people with rheumatic and musculoskeletal diseases; development and pilot testing. BMC Health Serv Res. 2019;19(1):265-12.
2. van Eijk-Hustings Y, Kroese M, Tan F, Boonen A, Bessems-Beks M, Landewé R. Challenges in demonstrating the effectiveness of multidisciplinary treatment on quality of life, participation and health care utilisation in patients with fibromyalgia: a randomised controlled trial. Clin Rheumatol. 2013;32(2):199-209.
3. Berdal G, Bø I, Dager TN, Dingsør A, Eppeland SG, Hagfors J, et al. Structured Goal Planning and Supportive Telephone Follow‐up in Rheumatology Care: Results From a Pragmatic, Stepped‐Wedge, Cluster‐Randomized Trial. Arthritis Care Res (Hoboken). 2018;70(11):1576-86.
4. Berdal G. Patient-specific goals and supportive follow-up in rheumatology rehabilitation. Analysis of content, summary of evidence, and evaluation of health effects. Doctoral Thesis. Institute of Health and Society: University of Oslo; 2019.
5. Grotle M, Klokkerud M, Kjeken I, Bremander A, Hagel S, Strömbeck B, et al. What's in the black box of arthritis rehabilitation? A comparison of rehabilitation practice for patients with inflammatory arthritis in northern Europe. J Rehabil Med. 2013;45(5):458-66.
6. Kjeken, I. The BRIDGE-trial: A multicentre stepped wedge randomised controlled trial to improve continuity and quality in rehabilitation for people with rheumatic and musculoskeletal diseases. 2017 (not published in detail. For the overarching presentation, see: National Library of Medicine: The BRIDGE Rehabilitation Trial (BRIDGE) 2017 [updated 02032020]. Available from: <https://clinicaltrials.gov/ct2/show/NCT03102814>. Accessed 8 May 2019).
7. The BRIDGE-trial research group: Sjekkliste (page 4-5), in *the BRIDGE-guide for helsepersonell* (Fidelity checklist, item no.1-25) (not published)
8. The BRIDGE-trial research group: Guiding booklets for patients and providers, respectively, in the BRIDGE-trial (not published)
9. Klokkerud M, Hagen KB, Kjeken I, Bremander A, Horslev-Petersen K, Vlieland TV, et al. Development of a framework identifying domains and elements of importance for arthritis rehabilitation. Journal of rehabilitation medicine. 2012;44(5):406-13.
10. Klokkerud M, Dagfinrud H, Uhlig T, Dager TN, Furunes KA, Klokkeide Å, et al. Developing and testing a consensus-based core set of outcome measures for rehabilitation in musculoskeletal diseases. Scand J Rheumatol. 2018;47(3):225-34.
